# Bioactive Polyketide and Diketopiperazine Derivatives from the Mangrove-Sediment-Derived Fungus *Aspergillus* sp. SCSIO41407

**DOI:** 10.3390/molecules26164851

**Published:** 2021-08-11

**Authors:** Jian Cai, Chunmei Chen, Yanhui Tan, Weihao Chen, Xiaowei Luo, Lianxiang Luo, Bin Yang, Yonghong Liu, Xuefeng Zhou

**Affiliations:** 1 CAS Key Laboratory of Tropical Marine Bio-Resources and Ecology, Guangdong Key Laboratory of Marine Materia Medica, South China Sea Institute of Oceanology, Chinese Academy of Sciences, Guangzhou 510301, China; caijian19@mails.ucas.ac.cn (J.C.); chenchunmei18@mails.ucas.ac.cn (C.C.); chenweihao17@mails.ucas.ac.cn (W.C.); yangbin@scsio.ac.cn (B.Y.); yonghongliu@scsio.ac.cn (Y.L.); 2 College of Earth and Planetary Sciences, University of Chinese Academy of Sciences, Beijing 100049, China; 3 Southern Marine Science and Engineering Guangdong Laboratory (Guangzhou), Guangzhou 511458, China; 4 State Key Laboratory for Chemistry and Molecular Engineering of Medicinal Resources, School of Chemistry and Pharmaceutical Sciences, Guangxi Normal University, Guilin 541004, China; tyh533@126.com; 5 Institute of Marine Drugs, Guangxi University of Chinese Medicine, Nanning 530200, China; luoxiaowei1991@126.com; 6 The Marine Biomedical Research Institute, Guangdong Medical University, Zhanjiang 524023, China; luolianxiang321@gdmu.edu.cn; 7 The Marine Biomedical Research Institute of Guangdong Zhanjiang, Zhanjiang 524023, China

**Keywords:** mangrove-sediment-derived fungus, *Aspergillus*, polyketides, diketopiperazines, NF-*κ*B, acetylcholinesterase

## Abstract

Ten polyketide derivatives (**1**–**10**), including a new natural product named (*E*)-2,4-dihydroxy-3-methyl-6-(2-oxopent-3-en-1-yl) benzaldehyde (**1**), and five known diketopiperazines (**11**–**15**), were isolated from the mangrove-sediment-derived fungus *Aspergillus* sp. SCSIO41407. The structures of **1**–**15** were determined via NMR and MS spectroscopic analysis. In a variety of bioactivity screening, **3** showed weak cytotoxicity against the A549 cell line, and **2** exhibited weak antibacterial activity against methicillin-resistant *Staphylococcus aureus* (MRSA). Compounds **3**, **5**, and **6** showed inhibition against acetylcholinesterase (AChE) with IC_50_ values of 23.9, 39.9, and 18.6 μM. Compounds **11**, **12**, and **14** exhibited obvious inhibitory activities of lipopolysaccharide (LPS)-induced nuclear factor-*κ*B (NF-*κ*B) with IC_50_ values of 19.2, 20.9, and 8.7 μM, and they also suppressed RANKL-induced osteoclast differentiation in bone marrow macrophages cells (BMMCs), with the concentration of 5 μM. In silico molecular docking with AChE and NF-*κ*B p65 protein were also performed to understand the inhibitory activities, and **1**, **11**–**14** showed obvious protein/ligand-binding effects to the NF-κB p65 protein.

## 1. Introduction

Mangrove is a unique ecosystem with high salinity, low pH value, and periodic tidal immersion, which is mainly distributed between the tropical and subtropical intertidal regions around the world and located at the junction of terrestrial and marine ecosystems. The variety and complexity of the mangrove soil environment leads to the diversity of soil microorganisms [1,2]. For a large number of microorganism resources, mangrove soil is an important source of lead compounds and antibiotics drugs. Mangrove-sediment-derived fungus can produce many novel bioactive compounds [3]. The fungus *Aspergillus* is the main part of the mangrove-sediment-derived fungi, which can produce bioactive compounds such as alkaloids [4], terpenoids [5], sterides [6], and polyketides [7]. These compounds have a variety of activities, including antibacterial [7], enzyme inhibitory [8], anti-inflammatory [9], antioxidant [10], antivirus [11], and cytotoxic activities [12]. The fungus *Aspergillus* is rich in metabolites, high in yield, and remarkable in biological activity, and it is the main source of new natural products from marine microorganisms. The search for active compounds and drug leads in marine-derived fungus *Aspergillus* has never stopped in the past two decades, and it is worth further exploring.

**Figure 1:**
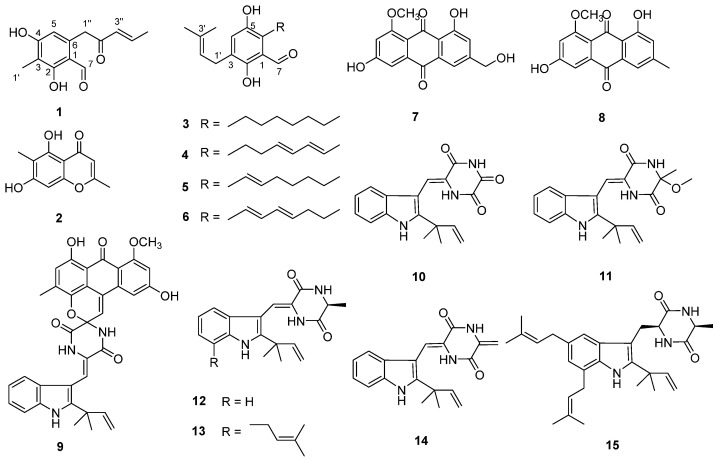
Chemical structures of **1**–**15**.

In our search for novel bioactive compounds from mangrove fungi, ten polyketides (**1**–**10**), including a new natural product (**1**), and five diketopiperazines (**11**–**15**) (Figure 1) were isolated and identified from a sediment-derived fungus *Aspergillus* sp. SCSIO 41407. Herein, their isolation, structure elucidation, and bioactivity are described in detail.

## 2. Results and Discussion

### 2.1. Structural Determination

**Figure 2:**
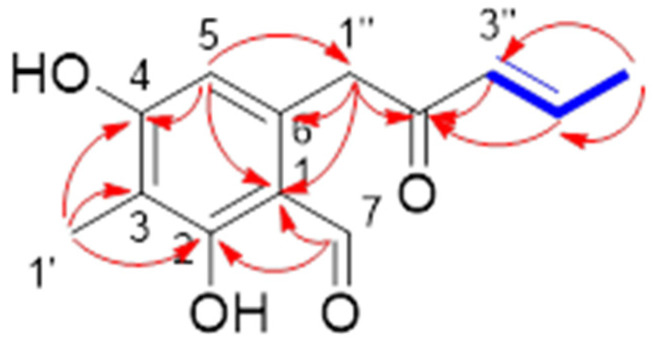
Key HMBC (red arrows) and ^1^H-^1^H COSY (blue bold line) correlations of **1**.

Compound **1** was isolated as tan crystalline solid, and its molecular formula was established as C_13_H_14_O_4_ according to the HRESIMS ion peak at *m*/*z* 235.0966 [M + H]^+^. The 1H NMR (Table 1) spectrum showed one aldehyde proton [9.80 (CH, s)], one aromatic proton [6.25 (CH, s)], two olefinic protons [6.28 (CH, d, *J* = 14.6 Hz), 7.09 (CH, m)], one methylene [4.15 (CH_2_, s)] and two methyls [2.03 (CH_3_, s), 1.95 (CH_3_, d, *J* = 6.7 Hz)]. The ^13^C NMR and DEPT data revealed the presence of 15 carbon signals, including six quaternary carbons (*δ*C 198.9, 165.2, 164.2, 139.5, 113.7, 111.4). Comparing the 1D NMR data with those of the literature [6], the structure of **1** was elucidated as (*E*)-2,4-dihydroxy-3-methyl-6-(2-oxopent-3-en-1-yl) benzaldehyde and confirmed via a 2D NMR experiment (Figure 2). Compound **1** appeared as an intermediate in the stereodivergent, chemoenzymatic synthesis of azaphilone natural products [13] and has been reported as a new natural product.

The known compounds were identified as eugenitol (**2**) [14], flavoglaucin (**3**) [15], isodihydroauroglaucin (**4**) [15], tetrahydroauroglaucin (**5**) [16], dihydroauroglaucin (**6**) [17], questinol (**7**) [18], questin (**8**) [11], variecolortide B (**9**) [19], neoechinulin E (**10**) [20], variecolorin H (**11**) [21], neoechinulin A (**12**) [22], variecolorin G (**13**) [21], neoechinulin B (**14**) [20], and echinulin (**15**) [23], via comparison of their NMR data with the reported literature data.

### 2.2. Cytotoxic and Antibacterial Activities

In the cytotoxicity screening test of **1**–**8** and **10**–**15** against A549 cell line, only **3** showed weak activity with an IC_50_ value of 22.2 μM. The antibacterial activities of **1**–**15** against six bacterial strains, *Acinetobacter baumannii* (ATCC 19606), *Staphylococcus aureus* (ATCC 29213), *Enterococcus faecalis* (ATCC 29212), *Klebsiella pneumoniae* (ATCC 13883), methicillin-resistant *Staphylococcus aureus* (MRSA), and *Escherichia coli* (ATCC 25922), were tested; however, only **2** exhibited weak antibacterial activity against MRSA with a MIC value of 485.4 μM (Table 2).

### 2.3. Acetylcholinesterase (AChE) Inhibitory Activities

All the isolated compounds (**1**–**15**) were evaluated for AChE inhibitory activity. Compounds **3**–**6** displayed inhibition against AChE with IC_50_ values of 23.9, 490.3, 39.9, and 18.6 μM, respectively, in comparison with the positive control orlistat (IC_50_ = 0.33 μM).

**Figure 3:**
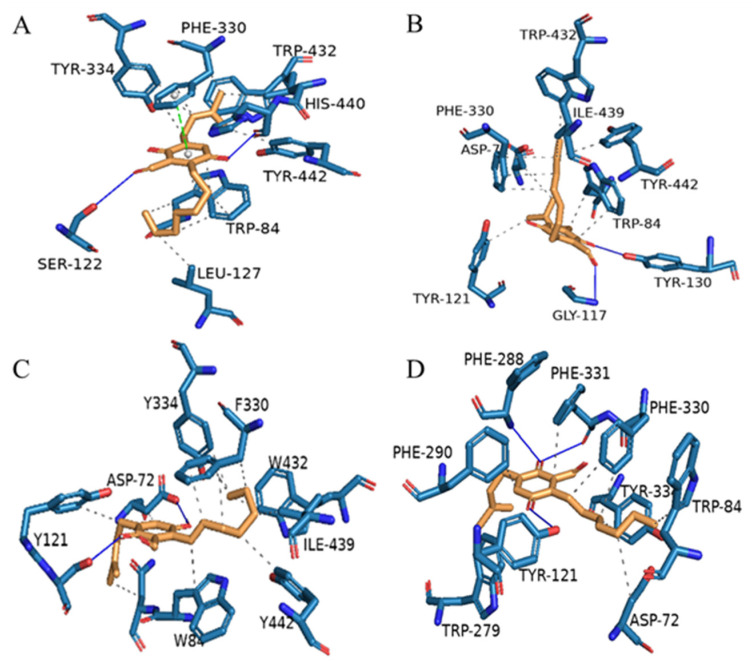
Proposed binding interactions of compounds **3** (**A**), **4** (**B**), **5** (**C**), and **6** (**D**) with the active site residues of AChE (PDB ID: 1UT6). Blue solid line: hydrogen bond; black dotted line: hydrophobic interaction; green dotted line: *π*-*π* stacking interaction.

In order to analyze the molecular interactions of the compounds, the selected active polyketide derivatives (**3**–**6**) were docked into the AChE (PDB: 1UT6) active site. As a result, those molecules fit comfortably into the binding pocket with similar binding positions, with negative binding free energy values (S value) from −8.15 to −8.44. Compounds **3**–**6** interacted with the AChE active site mainly through hydrogen bonds, *π*-*π* stacking contacts, and hydrophobic interactions (Figure 3). Most of the compounds’ hydrophobic interactions with the residues were similar, but hydrogen bonds were formed differently. 5-OH and aldehyde hydrogen of **3** formed hydrogen bonds with HIS-440 and SER-122, respectively. In addition, **3** also formed *π*-*π* stacking contacts with PHE-330. 2-OH and aldehyde hydrogen of **4** formed hydrogen bonds with TYR-130 and GLY-117, respectively. 2-OH and 5-OH of **5** formed hydrogen bonds with TYR-121 and ASP-72, respectively. In particular, **6** formed three hydrogen bonds, of which 2-OH formed two hydrogen bonds with PHE-288 and PHE-331, and 5-OH formed one hydrogen bond with Tyr-121. Nevertheless, all these interactions were beneficial for these compounds to anchor in the binding site of the enzyme.

### 2.4. Inhibition of Lipopolysaccharide (LPS)-Induced Nuclear Factor-κB (NF-κB)

Osteoclasts are unique multinucleated cells with bone-resorbing capacity, which are formed by the fusion of precursors from the bone marrow mononuclear macrophage lineage. Targeting osteoclast differentiation is a strategy for the treatment of osteolytic diseases. The differentiation and formation of osteoclasts are regulated by a variety of signal pathways, and the key pathways are induced by the receptor activator of NF-*κ*B ligand (RANKL), which is mainly secreted by bone cells. The binding of RANKL to its cognate receptor RANK causes a series of intracellular signaling events, such as the activation of NF-*κ*B. NF-*κ*B plays an important role in the differentiation and function of osteoclasts [24].

**Figure 4:**
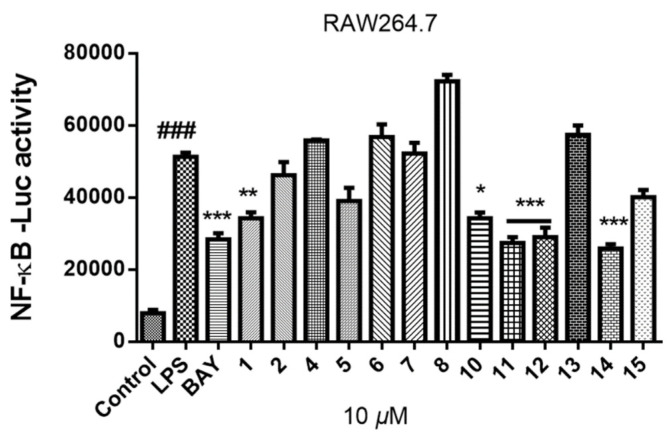
The inhibitory activities of compounds **1**–**8** and **10**–**15** on LPS-induced NF-*κ*B activation in RAW264.7 cells at 10 μM. All experiments were performed at least three times. The data are presented as the mean ± SD of representative experiments. ### *p* < 0.001 vs. control group; * *p* < 0.05, ** *p* < 0.01, *** *p* < 0.001 vs. LPS group.

In the process of screening osteoclast differentiation inhibitors from marine natural products, compounds **1**–**8** and **10**–**15** (10 μM) were primarily evaluated for their inhibitory activities of LPS-induced NF-*κ*B activation in RAW264.7 cells in this study (Figure 4). Compounds **1**, **10**–**12**, and **14** exhibited obvious inhibitory activities against LPS-induced NF-*κ*B, and the last three (**11**, **12**, and **14**) with IC_50_ values of 19.2, 20.9, and 8.7 μM, respectively.

**Figure 5:**
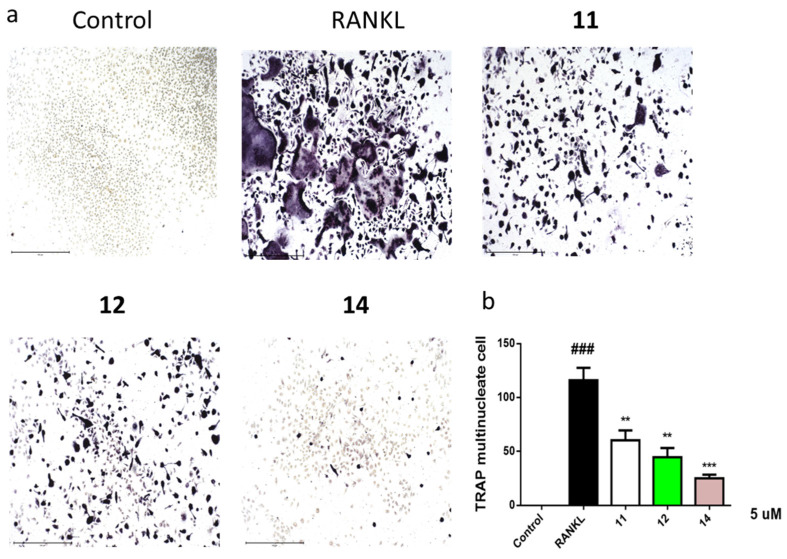
Compounds **11**, **12**, and **14** suppressed RANKL-induced osteoclast differentiation. Representative images of osteoclasts from BMMCs treated with **11**, **12**, and **14** (5 μM) for 3 days; tartrate-resistant acidic phosphatase (TRAP)-positive multinucleated cells were regarded as osteoclasts (**a**) and quantified (**b**). All experiments were performed at least three times. The data are presented as the mean ± SD of representative experiments. ### *p* < 0.001 vs. control group; ** *p* < 0.01, *** *p* < 0.001 vs. RANKL group.

Diketopiperazines **11**, **12**, and **14** were further evaluated for their effects on RANKL-induced osteoclastogenesis, and the results showed that **11**, **12**, and **14** at 5 μM suppressed RANKL-induced osteoclast differentiation in bone marrow macrophages cells (BMMCs) (Figure 5a,b). Consequently, these compounds could be promising osteoclast differentiation inhibitors for the treatment of osteoclast-related diseases.

**Figure 6:**
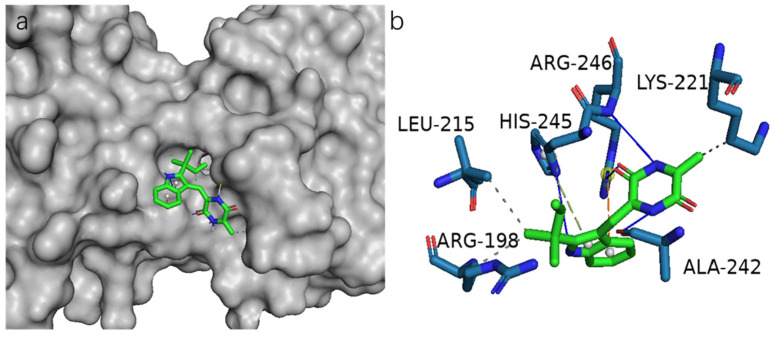
Molecular docking result of compound **14** in the NF-*κ*B p65 protein (PDB ID: 1MY5). (**a**) Binding sites of **14** with NF-*κ*B p65. (**b**) 3D diagram of the binding interactions of **14** with the active site residues of the NF-*κ*B p65 receptor. Blue solid line: hydrogen bond; grey dotted line: hydrophobic interaction; green dotted line: *π*-*π* stacking interaction; orange dotted line: *π*-cation interaction.

To further evaluate NF-*κ*B as an important molecular target for **1**, **11**–**14** to inhibit osteoclastogenesis, we performed a docking study to analyze the possible binding of these compounds and NF-*κ*B p65 protein (PDB ID: 1MY5). As a result, **14** bound remarkably to the active site of NF-*κ*B p65 as supported by the 3D structure of NF-*κ*B p65 docked with **14** (Figure 6a). The hydroxyl at N-11 and the carbonyl group at C-10 of **14** formed hydrogen bonds with the residue Arg-246, whereas the hydroxyl at N-1 and N-14 formed hydrogen bonds with the residue Arg-245 and Ala-242, respectively. In addition, **14** also formed *π*-*π* stacking, *π*-cation, and hydrophobic interactions with the NF-*κ*B p65 (Figure 6b). Compound **14** showed the strongest binding to the NF-*κ*B p65 protein after analysis. The binding activity of **14** was also in line with their effects in the osteoclastic TRAP assay. The new natural product **1** with 10 μM also showed obvious inhibitory activities against LPS-induced NF-κB in bioassay. In the docking study, **1** formed six hydrogen bonds and three hydrophobic interactions (Figure S38B). The hydroxyl at C-4 and C-2 of **1** formed hydrogen bonds with the residue LYS-221. The hydroxyl at C-2 also formed hydrogen bonds with the residues VAL-219 and GLN-247. And the carbonyl group at C-2’ formed two hydrogen bonds with the residue ARG-246. According to the analysis of all docking results, it can be inferred that the residues Arg-246 and Lys-221 are the dominant active sites. Compounds **11**–**13** also showed obvious protein/ligand-binding effects to the NF-κB p65 protein. Docking studies of compounds **1**, **11**–**13** on NF-*κ*B p65 are presented in Figure S38.

## 3. Materials and Methods

### 3.1. General Experimental Procedures

Optical rotations were measured on a PerkinElmer MPC 500 (PerkinElmer, Waltham, MA, USA) polarimeter. The UV spectra were obtained with a Shimadzu UV-2600 PC spectrometer (Shimadzu, Kyoto, Japan). IR spectra were determined with an IR Affinity-1 spectrometer (Shimadzu, Kyoto, Japan). NMR spectra were recorded on a Bruker Avance spectrometer (Bruker, Billerica, MA, USA) operating at 500 MHz and 700 MHz for ^1^H NMR and 125 MHz and 175 MHz for ^13^C NMR used tetramethylsilane as an internal standard. HRESIMS spectra were acquired on a Bruker miXis TOF-QII mass spectrometer (Bruker, Billerica, MA, USA). TLC and column chromatography (CC) were performed on plates precoated with silica gel GF254 (10−40 μm) and over silica gel (200–300 mesh) (Qingdao Marine Chemical Factory, Qingdao, China), respectively. Spots were detected on TLC (Qingdao Marine Chemical Factory, Qingdao, China) under 254 nm UV light. All solvents employed were analytical grade (Tianjin Fuyu Chemical and Industry Factory, Tianjin, China). Semipreparative HPLC was performed using an ODS column (YMC-pack ODS-A, YMC Co., Ltd., 10 × 250 mm, 5 μm, Kyoto, Japan). Artificial sea salt was a commercial product (Guangzhou Haili Aquarium Technology Company, Guangzhou, China).

### 3.2. Fungal Material

The fungal strain *Aspergillus* sp. SCSIO41407 was isolated from a mangrove sediment sample, collected in the Hongsha River estuary to the South China Sea, in Sanya city, Hainan Island, and identified according to the ITS region sequence data of the rDNA (Supplementary Materials). The strain was stored on Muller Hinton broth (MB) agar (malt extract 15 g, sea salt 10 g, agar 15 g, H_2_O 1 L, pH 7.4–7.8) at 4 °C and deposited in the CAS Key Laboratory of Tropical Marine Bioresources and Ecology, South China Sea Institute of Oceanology, Chinese Academy of Sciences, Guangzhou, China.

### 3.3. Fermentation, Extraction and Isolation

The strain *Aspergillus* sp. SCSIO41407 was cultured in 4 × 500 mL erlenmeyer flasks, each containing 200 mL of the seed medium (malt extract 15 g, sea salt 10 g, H_2_O 1 L, pH 7.4–7.8) for 3 days at 28 °C on a rotating shaker (180 rpm), which was then transferred into 120 × 1000 mL Erlenmeyer flasks, each containing 300 mL liquid medium (3% sea salt, 2% mannitol, 1% glucose, 1% monosodium glutamate, 2% maltose, 0.05% KH_2_PO_4_, 0.3% yeast extract, 0.1% corn steep liquor, 0.03% MgSO_4_·7H_2_O, pH = 7.5) for 11 days at 28 °C on a rotating shaker (180 rpm). The fermented cultures were overlaid and extracted with EtOAc three times to afford an organic extract (34.9 g). The EtOAc crude extract was chromatographed over a silica gel column eluted with PE-CH_2_Cl_2_-MeOH (50:1:0 to 0:0:1, *v*/*v*) in a gradient to yield of eight fractions (Frs.1–8). Fr.1 was divided into six fractions (Fr1.1–6) via ODS silica gel chromatography eluting with MeOH/H_2_O (10%–100%). Fr.1.1 was separated using semi-preparative HPLC (82% MeCN/H_2_O, 2 mL/min) to afford **3** (5.7 mg, t*_R_* = 28.9 min), **5** (2.3 mg, t*_R_* = 27.6 min), and **4** (3.1 mg, t*_R_* = 18.5 min). Fr.1.2 was purified using semi-preparative HPLC (90%MeOH/H_2_O, 2 mL/min) to afford **6** (5.3 mg, t*_R_* = 15.2 min). Fr.3 was separated using semi-preparative HPLC (42%MeCN/H_2_O, 2 mL/min) to afford **1** (8.1 mg, t*_R_* = 22.0 min) and **2** (3.8 mg, t*_R_* = 18.0 min). Fr.4 was divided into five fractions (Fr4.1–5) via ODS silica gel chromatography eluting with MeOH/H_2_O (10%–100%). Fr.4.3 was separated using semi-preparative HPLC (60% MeOH/H_2_O, 2 mL/min) to afford **11** (3.5 mg, t*_R_* = 16.3 min) and **14** (4.2 mg, t*_R_* = 19.5 min). Fr.4.4 was separated via semi-preparative HPLC (75%MeCN/H_2_O, 2 mL/min) to yield **8** (2.9 mg, t*_R_* = 14.1 min) and **13** (3.9 mg, t*_R_* = 11.2 min). Fr.5 was separated via semipreparative HPLC (60%MeOH/H2O, 2 mL/min) to yield **12** (5.0 mg, t*_R_* = 27.5 min) and **10** (6.7 mg, t*_R_* = 19.5 min). **15** (2.9 mg, t*_R_* = 16.1 min) was purified from Fr.6 (90%MeOH/H_2_O, 2 mL/min). Fr.8 was further divided into three fractions (Fr.8.1–Fr.8.3) using silica gel chromatography eluting with EtOAc/PE (5–100%). Compound **9** (1.9 mg, t*_R_* = 13.9 min) was purified from Fr.8.1 (65%MeOH/H_2_O, 3 mL/min) and **7** (4.5 mg, t*_R_* = 29.0 min) was isolated from Fr.8.2 (60%MeOH/H_2_O, 2 mL/min) via semipreparative HPLC.

#### (E)-2,4-Dihydroxy-3-methyl-6-(2-oxopent-3-en-1-yl) benzaldehyde (**1**)

Tan crystalline solid; UV (MeOH) *λ*max (log*ε*) 218 (3.99), 295 (3.73) nm; IR (film) *ν*max 3250, 2927, 1622 cm^−1^; ^1^H and ^13^C NMR spectral data, see Table 1; HRESIMS *m*/*z* 235.0966 [M + H]^+^ (calcd for C_13_H_15_O_4_^+^ 235.0965).

### 3.4. Bioactivity Assay

AChE inhibitory activity was evaluated in vitro according to the modified Ellman method [25]. Briefly, 0.1 U/mL AChE solution was prepared by dissolving in phosphate buffer (pH 8.0). The test sample and enzyme buffer were mixed in 96-well plates and incubated for 20 min at 30 °C. Then, 5,5′-dithiobis (2-nitrobenzoic acid) and acetylthiocholine iodide were added, and the enzyme reaction was allowed to proceed for 30 min at 30 °C. AChE activity was determined by measuring the degradation of acetylthiocholine iodide to thiocholine and acetic acid at 405 nm using a microplate reader.

The inhibitory activities of LPS-induced NF-*κ*B activation in RAW264.7 cells was evaluated as detected by luciferase reporter gene assay as described previously [18]. In brief, the RAW264.7 cells stably transfected with a luciferase reporter gene were plated in 96-well plates and then pretreated with testd compounds (10 μM) and BAY11-7082 (NF-*κ*B inhibitor as positive control, 5 μM, Sigma-Aldrich) for 30 min, followed by 5 μg/mL LPS stimulation for 8 h. Cells were harvested, and luciferase activities of the triplicate tests were measured using the luciferase assay system (Promega, Madison, WI, USA).

For further study of **11**, **12**, and **14** on osteoclastogenesis, BMMCs were added with macrophage-stimulating factor (50 ng/mL) and RANKL (100 ng/mL) stimulation at 5 μM concentration for 3 days. Then, the cells were fixed and stained for TRAP activity and the images were photographed using an inverted microscope (Nikon, Tokyo, Japan). Data are expressed as the mean ± SD and analyzed using GraphPad Prism 7.0 software (GraphPad, San Diego, CA, USA). Statistical differences among groups were performed using one-way analysis of variance with a Bonferroni post-hoc test. A *p*-value of < 0.05 was considered statistically significant.

The cytotoxicity of **1**–**8** and **10**–**15** against the A549 cell line was preliminarily evaluated via the MTT method as reported in our previous study [26], and the antibacterial activities of **1**–**15** against six bacterial strains, *Acinetobacter baumannii* (ATCC 19606), *Staphylococcus aureus* (ATCC 29213), *Enterococcus faecalis* (ATCC 29212), *Klebsiella pneumoniae* (ATCC 13883), MRSA, and *Escherichia coli* (ATCC 25922), were evaluated using a modification of the broth microdilution method [27]. Gentamycin was used as the positive control against MRSA with a MIC value of 1.4 μM.

### 3.5. Molecular Docking

Molecular docking simulation was performed using the software AutoDock Tools (ADT 1.5.6) [28]. The crystal structure of AChE from *Tetronarce californica* (PDB ID: 1UT6) [29] and NF-*κ*B p65 (PDB: 1MY5) [24] were obtained from the Protein Data Bank (http://www.rcsb.org, accessed on 5 July 2021). The structures as ligands were generated in ChemBioOffice 18.0, followed by an MM2 calculation to minimize the conformation energy. The sizes of the grid boxes were set according to the literature [24,29]. The other docking parameters, settings, and calculations were as default, and the docking results were analyzed in the software PyMOL 2.4.0.

## 4. Conclusions

In summary, chemical investigation of the mangrove-sediment-derived fungus *Aspergillus* sp. SCSIO41407 led to the isolation of a new natural product, (*E*)-2,4-dihydroxy-3-methyl-6- (2-oxopent-3-en-1-yl) benzaldehyde (**1**), together with nine known polyketide derivatives (**2**–**10**) and five known diketopiperazines (**11**–**15**). In a variety of bioactivity screening, **3** showed weak cytotoxicity against the A549 cell line, and **2** exhibited weak antibacterial activity against MRSA. Compounds **3**, **5**, and **6** showed inhibition against AChE. Compounds **11**, **12**, and **14** exhibited obvious inhibition of LPS-induced NF-*κ*B and suppressed RANKL-induced osteoclast differentiation in BMMCs at 5 μM. In silico molecular docking with AChE and NF-κB p65 protein were also performed to understand the inhibitory activities.

In this study, the new natural benzaldehyde derivative (**1**) and several diketopiperazines (such as **11**, **12**, and **14**) have attracted our attention with their inhibitory activities against LPS-induced NF-κB, and they deserve further study as potential osteoclast differentiation inhibitors. The mangrove-sediment-derived fungus *Aspergillus* sp. SCSIO41407 was uncovered with productive bioactive metabolites. It is supported that mangrove-sediment-derived fungi are important sources of drug lead compounds.

## Figures and Tables

**Table 1:** NMR data (500 and 125 MHz, CD_3_OD, *δ*H/ppm) for **1**.

No.	*δ*_C_	*δ*_H_ (*J* in Hz)	HMBC	COSY
1	113.7			
2	165.2			
3	111.4			
4	164.5			
5	111.7	6.25 (CH, s)	C-1, C-1″, C-4	
6	139.5			
7	194.8	9.80 (CH, s)	C-2, C-1, C-5	
1′	7.2	2.03 (CH_3_, s)	C-4, C-2, C-3	
1″	43.9	4.15 (CH_2_, s)	C-2″, C-6, C-1, C-5	
2″	198.9			
3″	131.8	6.28 (CH, d, *J* = 14.6 Hz)	C-5″, 2″	H-4″, H-5″
4″	146.3	7.09 (CH, m)	C-2″, C-5″	H-5″, H-3″
5″	18.5	1.95 (CH_3_, d, *J* = 6.7 Hz)	C-4″, C-3″	C-4″, C-3″

**Table 2:** Cytotoxic and antibacterial activities of **1**–**8** and **10**–**15**.

Compounds	Cytotoxic Activities	Antibacterial Activities	
	A549 (IC_50_, μM)	MRSA (MIC, μM)	* Other Bacterial Strains
2	/	485.4 μM	/
3	22.2 μM	/	/
1, 4–8, 10–15	/	/	/

/: No obvious activities; * Acinetobacter baumannii (ATCC 19606), Staphylococcus aureus (ATCC 29213), Enterococcus faecalis (ATCC 29212), Klebsiella pneumoniae (ATCC 13883), and Escherichia coli (ATCC 25922).

## Data Availability

All data and figures in this study are openly available.

## Supplementary Materials

The following are available online, Figures S1–S9, S10–S37: 1H, ^13^C-NMR, HSQC, COSY, HMBC, UV, IR, and HRESIMS spectra of compound **1**, ^1^H and ^13^C-NMR spectra of compounds **2**–**15**. Figure S38: Molecular docking of **1**, **10**–**12** with NF-*κ*B p65.

## Sample Availability

Samples of the compounds **1**–**15** are available from the authors.
