# Supplementary material for: Bioactive Polyketide and Diketopiperazine Derivatives from the Mangrove-Sediment-Derived Fungus Aspergillus sp. SCSIO41407
Source: Molecules. 2021 Aug 11;26(16):4851. doi: 10.3390/molecules26164851 (PMC8399180; doi:10.3390/molecules26164851)
Supplement: Supplementary file 1 [file molecules-26-04851-s001.zip › molecules-1316045-supplementary.pdf]

Supplementary Materials

Bioactive polyketide and diketopiperazine derivatives from the mangrove-sediment-derived fungus *Aspergillus* sp. SCSIO41407

Jian Cai [a,b,c], Chunmei Chen [a,b], Yanhui Tan [d], Weihao Chen [a,b], Xiaowei Luo [e], Lianxiang Luo [f,g], Bin Yang [a,b,c], Yonghong Liu [a,b,c], Xuefeng Zhou [a,b,c,]*

[a] CAS Key Laboratory of Tropical Marine Bio-resources and Ecology, Guangdong Key Laboratory of Marine Materia Medica, South China Sea Institute of Oceanology, Chinese Academy of Sciences, Guangzhou, 510301, China;

[b] University of Chinese Academy of Sciences, Beijing, 100049, China

[c] Southern Marine Science and Engineering Guangdong Laboratory (Guangzhou), Guangzhou, 511458, China;

[d] State Key Laboratory for Chemistry and Molecular Engineering of Medicinal Resources, School of Chemistry and Pharmaceutical Sciences, Guangxi Normal University, Guilin, 541004, China;

[e] Institute of Marine Drugs, Guangxi University of Chinese Medicine, Nanning 530200, China;

[f] The Marine Biomedical Research Institute, Guangdong Medical University, Zhanjiang, 524023, China;

[g] The Marine Biomedical Research Institute of Guangdong Zhanjiang, Zhanjiang, 524023, China.

*Correspondence: xfzhou@scsio.ac.cn; Tel: +86-020-89023174.

Figure S1. ^{1}H NMR spectrum of **1** (CD_3OD, 500 MHz).
Figure S2. ^{1}C NMR spectrum of **1** (CD_3OD, 500 MHz).
Figure S3. The DPET spectrum of **1** (CD_3OD).
Figure S4. HSQC spectrum of **1** (CD_3OD).
Figure S5. HMBC spectrum of **1** (CD_3OD).
Figure S6. COSY spectrum of **1** (CD_3OD).
Figure S7. The UV spectrum of **1**.
Figure S8. The IR spectrum of **1**.
Figure S9. HRESIMS spectrum of compound **1**.
Figure S10. ^{1}H NMR spectrum of **2** (DMSO-d_6, 700MHz).
Figure S11. ^{1}C NMR spectrum of **2** (DMSO-d_6, 175 MHz).
Figure S12. ^{1}H NMR spectrum of **3** (DMSO-d_6, 500 MHz).
Figure S13. ^{1}C NMR spectrum of **3** (DMSO-d_6, 125 MHz).
Figure S14. ^{1}H NMR spectrum of **4** (DMSO-d_6, 700 MHz).
Figure S15. ^{1}C NMR spectrum of **4** (DMSO-d_6, 175 MHz).
Figure S16. ^{1}H NMR spectrum of **5** (DMSO-d_6, 700 MHz).
Figure S17. ^{1}C NMR spectrum of **5** (DMSO-d_6, 175 MHz).

Figure S18. ^{1}H NMR spectrum of **6** ($CDCl_3$, 700 MHz).
Figure S19. ^{1}C NMR spectrum of **6** ($CDCl_3$, 175 MHz).
Figure S20. ^{1}H NMR spectrum of **7** (CD_3OD, 700 MHz).
Figure S21. ^{1}C NMR spectrum of **7** (CD_3OD, 175 MHz).
Figure S22. ^{1}H NMR spectrum of **8** (DMSO-d_6, 700 MHz).
Figure S23. ^{1}C NMR spectrum of **8** (DMSO-d_6, 175 MHz).
Figure S24. ^{1}H NMR spectrum of **9** (DMSO-d_6, 700 MHz).
Figure S25. ^{1}C NMR spectrum of **9** (DMSO-d_6, 175 MHz).
Figure S26. ^{1}H NMR spectrum of **10** (DMSO-d_6, 500 MHz).
Figure S27. ^{1}C NMR spectrum of **10** (DMSO-d_6, 125 MHz).
Figure S28. ^{1}H NMR spectrum of **11** (DMSO-d_6, 700 MHz).
Figure S29. ^{1}C NMR spectrum of **11** (DMSO-d_6, 175 MHz).
Figure S30. ^{1}H NMR spectrum of **12** (DMSO-d_6, 500 MHz).
Figure S31. ^{1}C NMR spectrum of **12** (DMSO-d_6, 125 MHz).
Figure S32. ^{1}H NMR spectrum of **13** (DMSO-d_6, 700 MHz).
Figure S33. ^{1}C NMR spectrum of **13** (DMSO-d_6, 175 MHz).
Figure S34. ^{1}H NMR spectrum of **14** (DMSO-d_6, 700 MHz).
Figure S35. ^{1}C NMR spectrum of **14** (DMSO-d_6, 175 MHz).
Figure S36. ^{1}H NMR spectrum of **15** (DMSO-d_6, 500 MHz).
Figure S37. ^{1}C NMR spectrum of **15** (DMSO-d_6, 125 MHz).
Figure S38. Molecular docking of **1**, **10**, **11**, and **12** with NF-*κ*B p65.
ITS sequence of the strain *Aspergillius* sp. SCSIO41407.

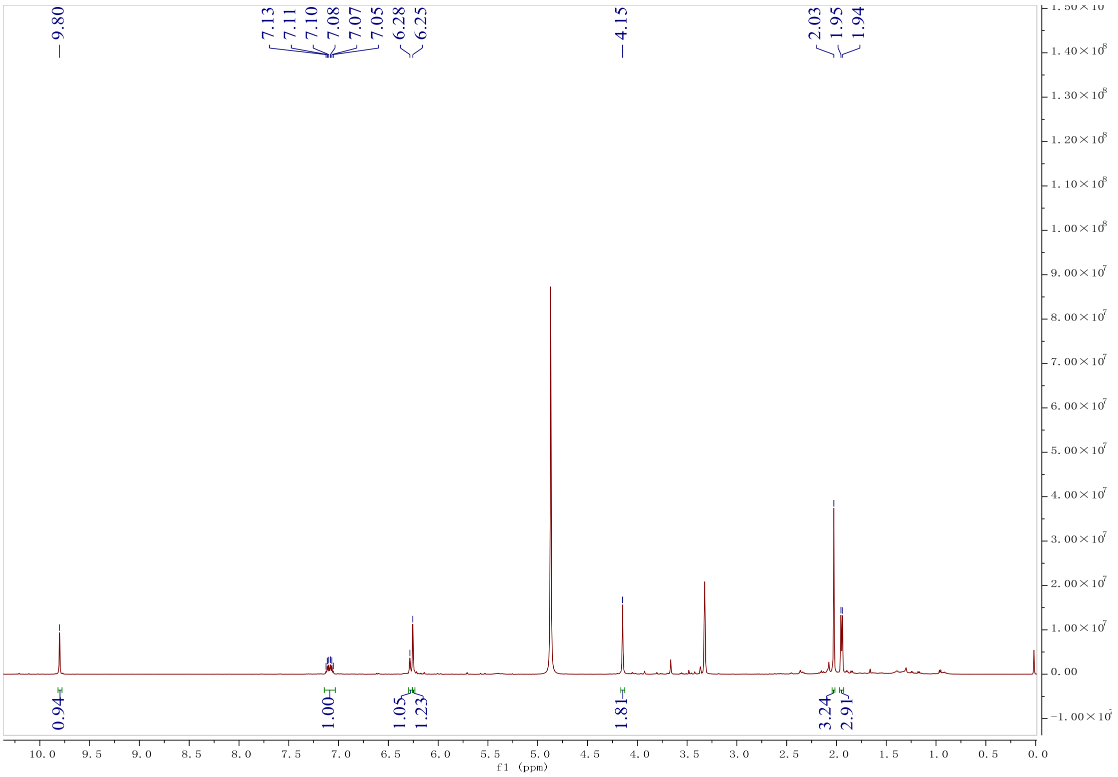

Figure S1. ^{1}H NMR spectrum of **1** (CD_3OD, 500 MHz).

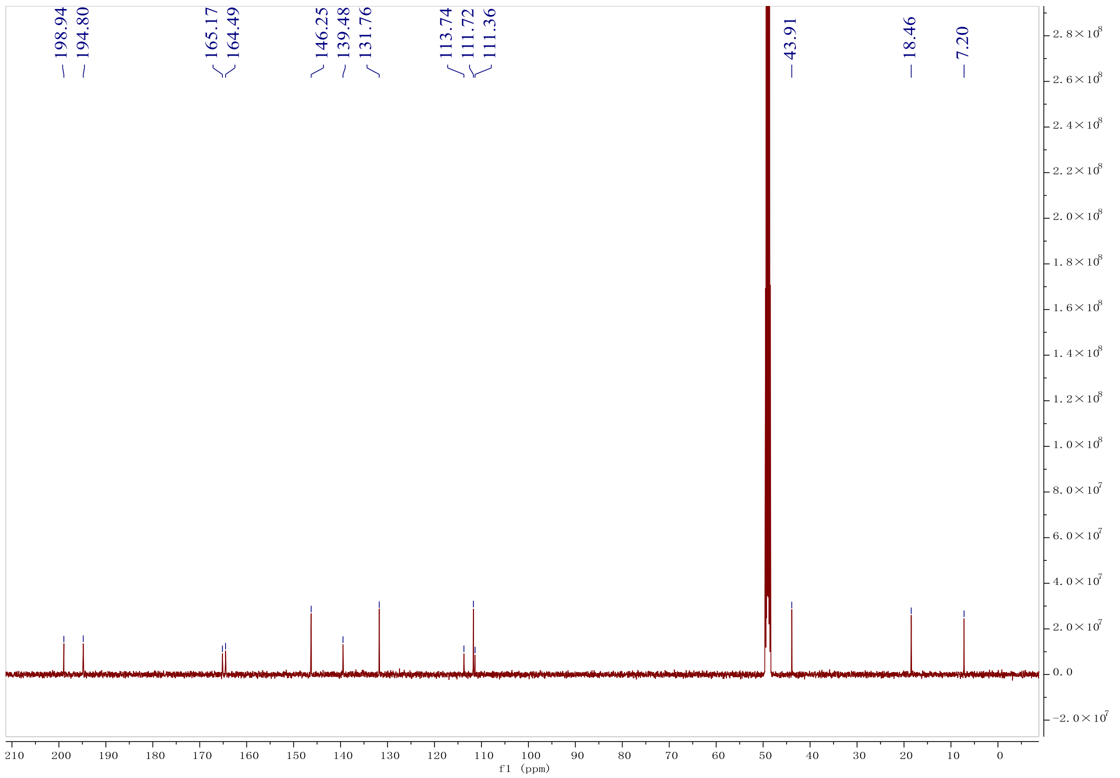

Figure S2. ^{1}C NMR spectrum of **1** (CD_3OD, 125 MHz).

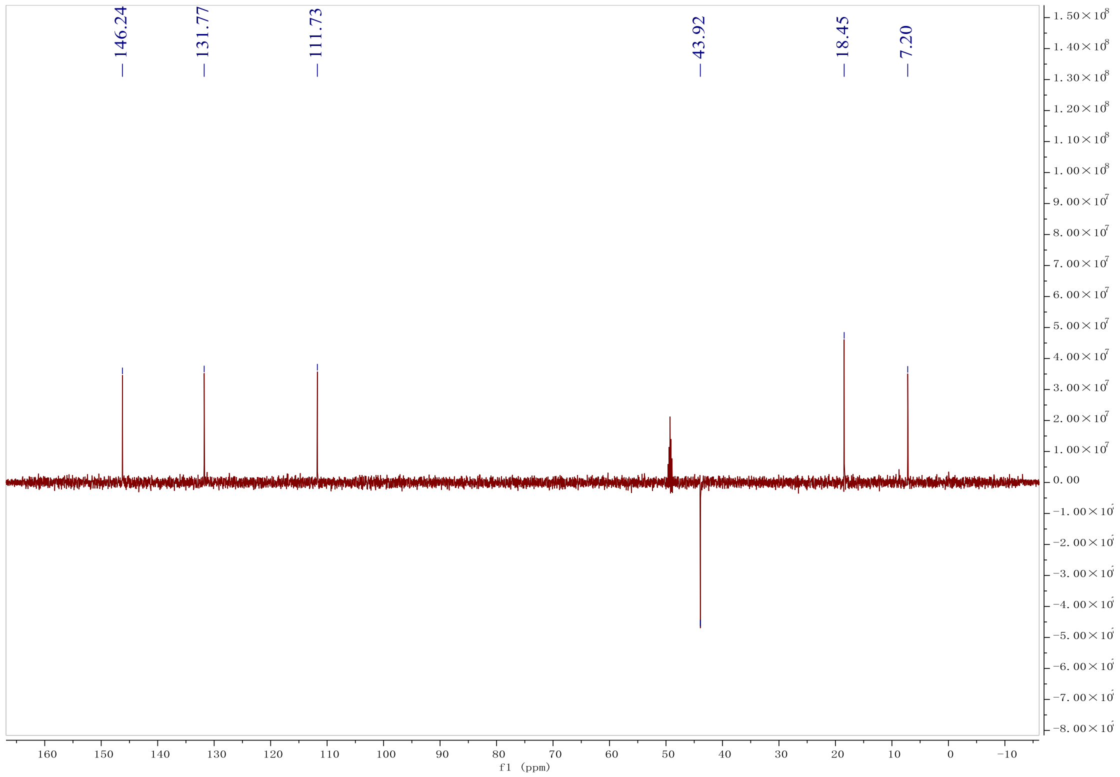

Figure S3. The DPET spectrum of **1** (CD_3OD).

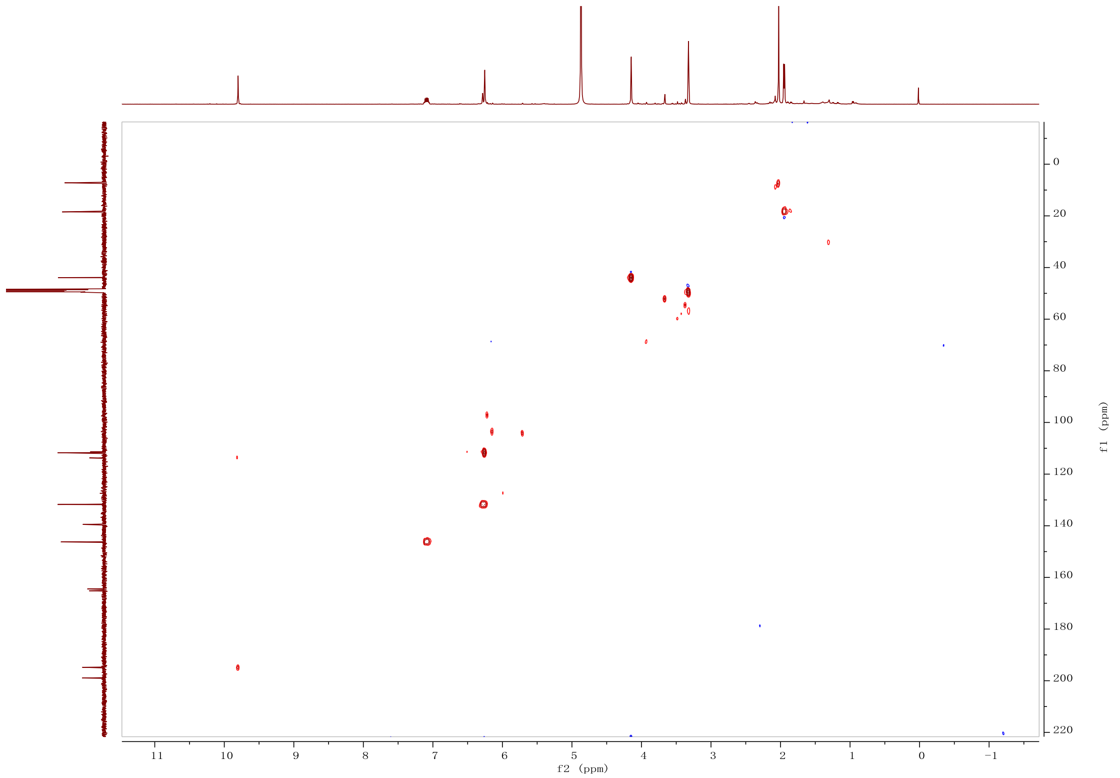

Figure S4. HSQC spectrum of **1** (CD_3OD).

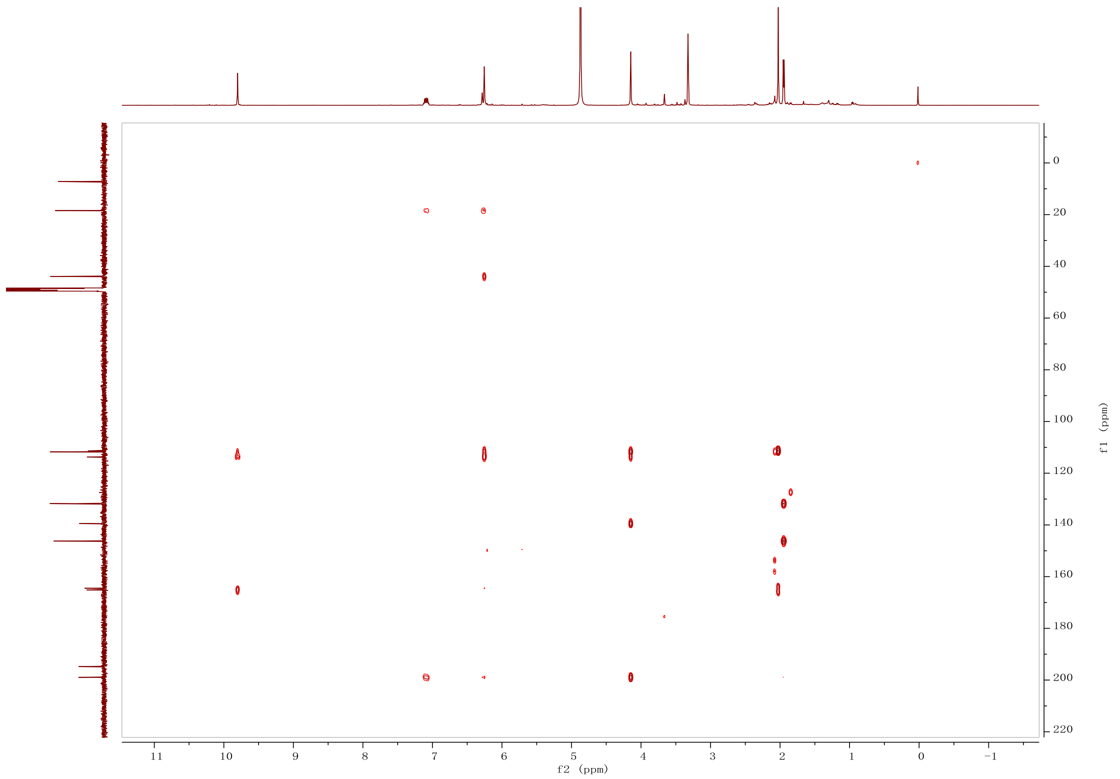

Figure S5. HMBC spectrum of **1** (CD_3OD).

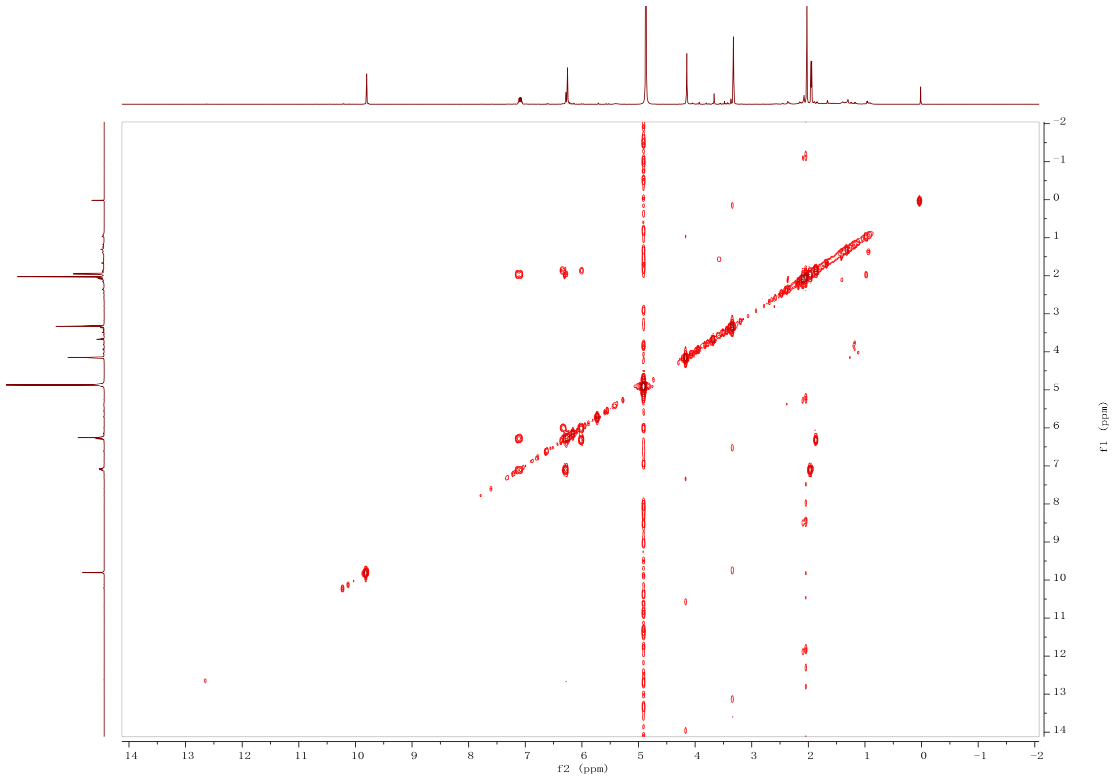

Figure S6. COSY spectrum of **1** (CD_3OD).

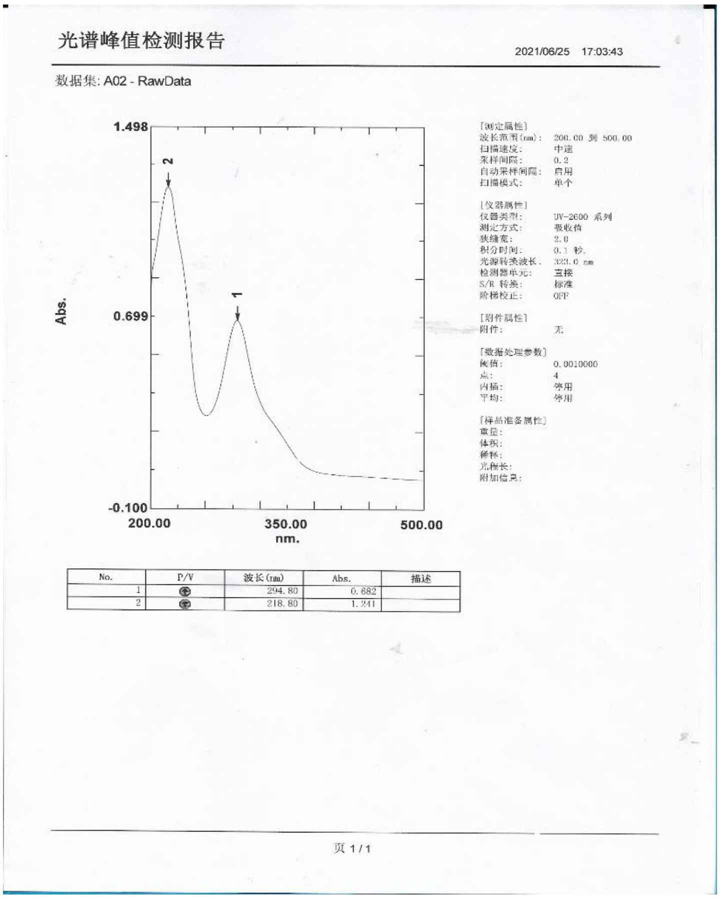

光谱峰值检测报告

2021/06/25 17:03:43

数据集: A02 - RawData

[测定属性]
波长范围(nm): 200.00 到 500.00
扫描速度: 中速
采样间隔: 0.2
自动采样间隔: 启用
扫描模式: 单个

[仪器属性]
仪器类型: UV-2600 系列
测定方式: 吸收值
狭缝宽: 2.0
积分时间: 0.1 秒.
光源转换波长. 323.0 nm
检测器单元: 直接
S/R 转换: 标准
阶梯校正: OFF

[附件属性]
附件: 无

[数据处理参数]
阈值: 0.0010000
点: 4
内插: 停用
平均: 停用

[样品准备属性]
重量:
体积:
稀释:
光程长:
附加信息:

No.	P/V	波长(nm)	Abs.	描述
1	[illegible]	294.80	0.682	
2	[illegible]	218.80	1.241	

页 1 / 1

Figure S7. The UV spectrum of **1**.

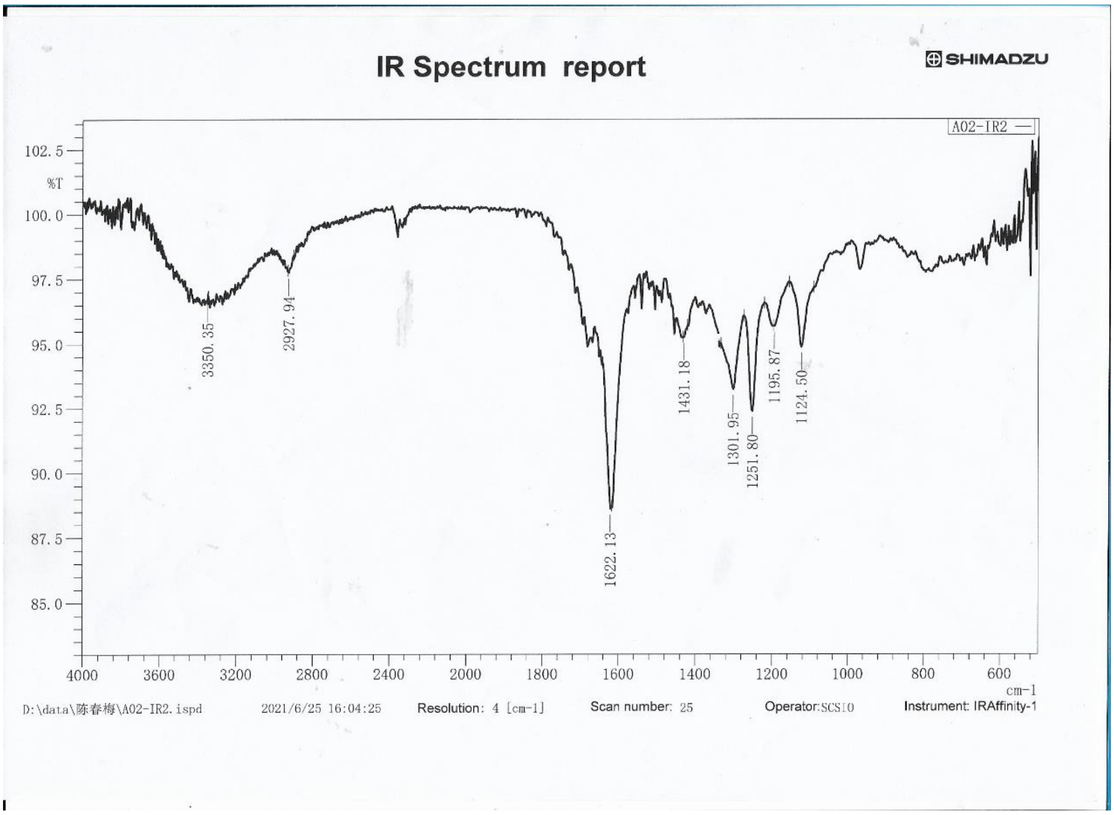

Figure S8. The IR spectrum of **1**.

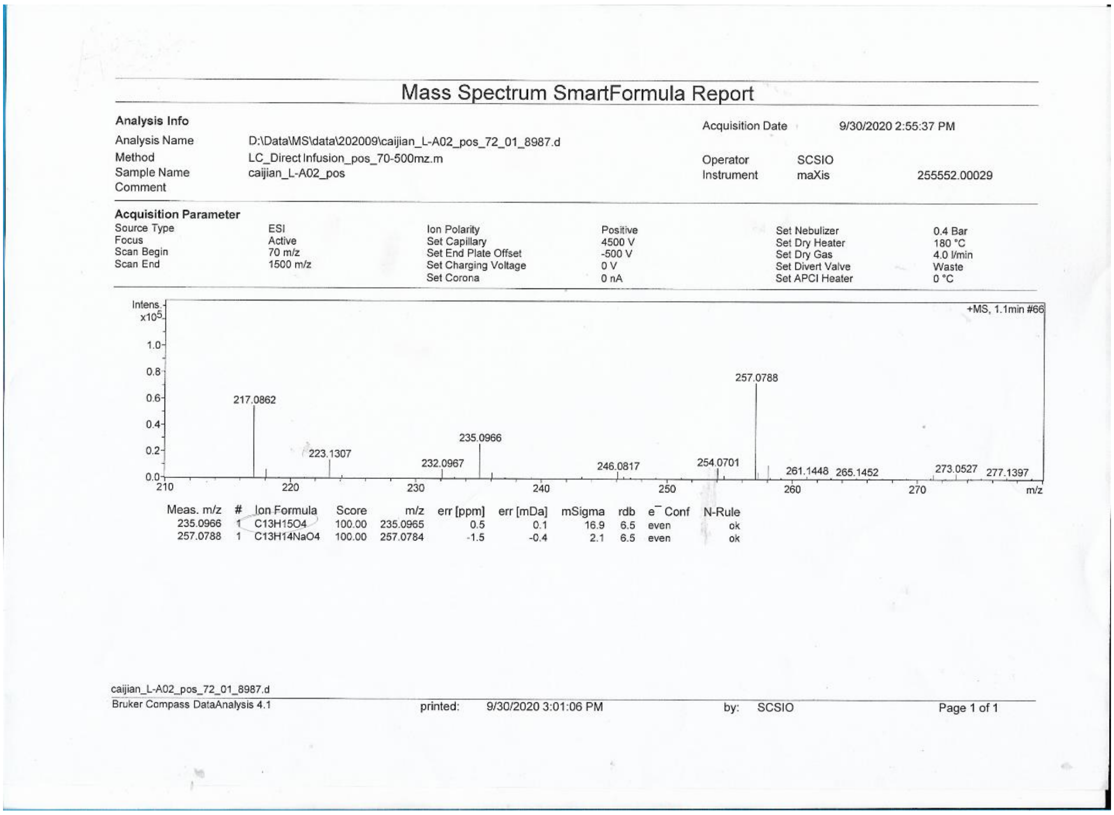

Figure S9. HRESIMS spectrum of compound 1.

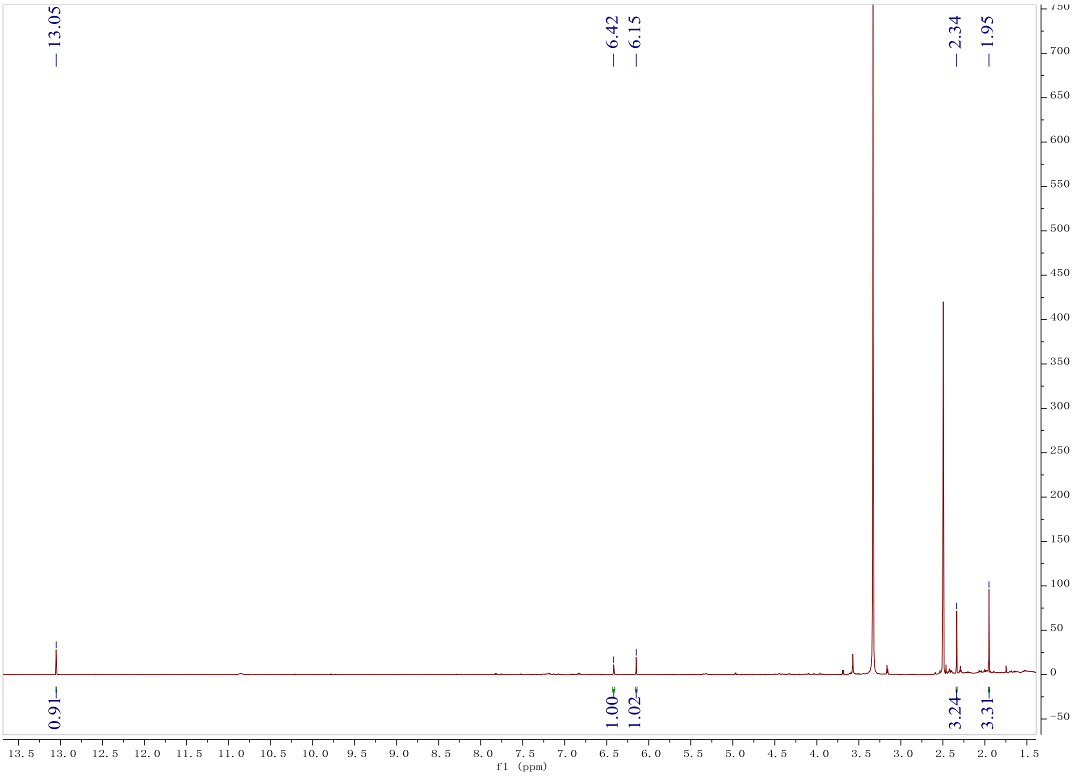

Figure S10. ^{1}H NMR spectrum of **2** ((DMSO-d_6, 700 MHz).

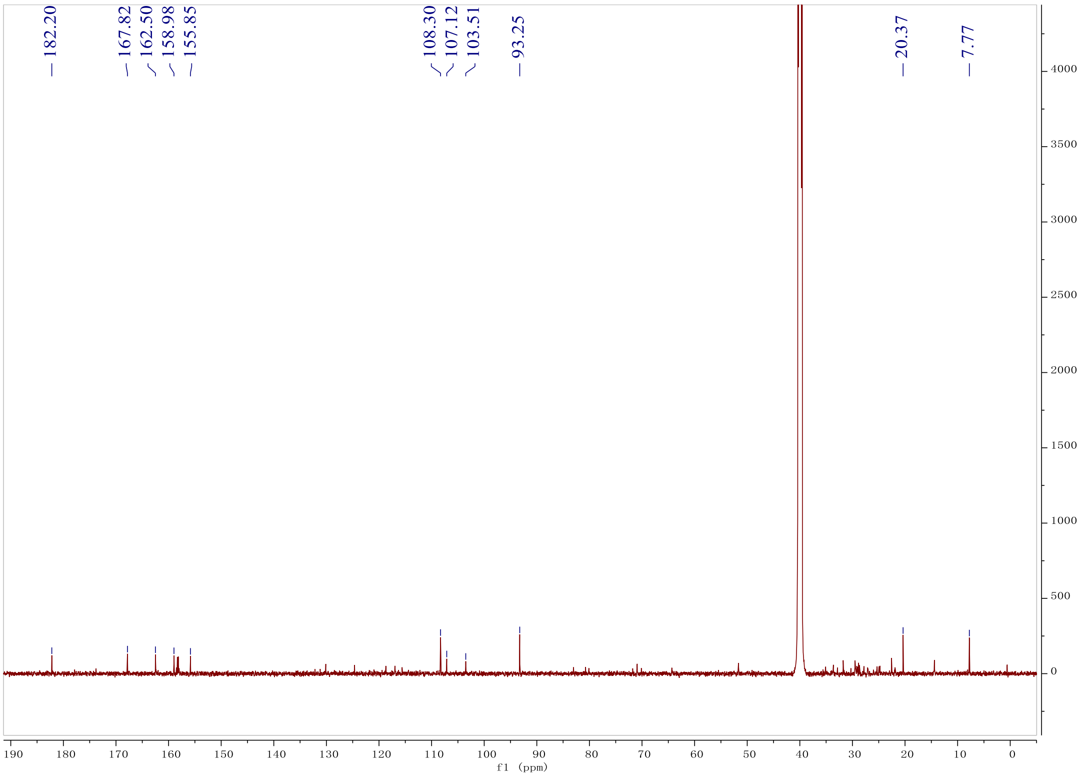

Figure S11. ^{1}C NMR spectrum of **2** ((DMSO-d_6, 175 MHz).

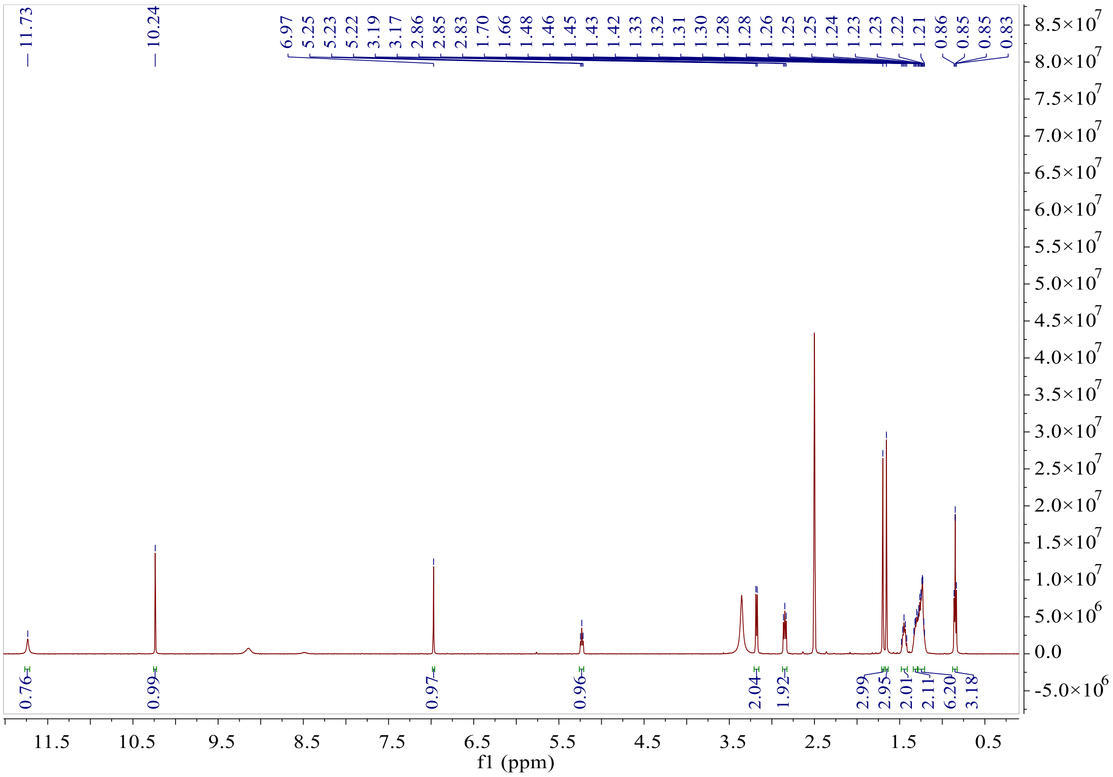

Figure S12. ^{1}H NMR spectrum of **3** ((DMSO-d_6, 500 MHz).

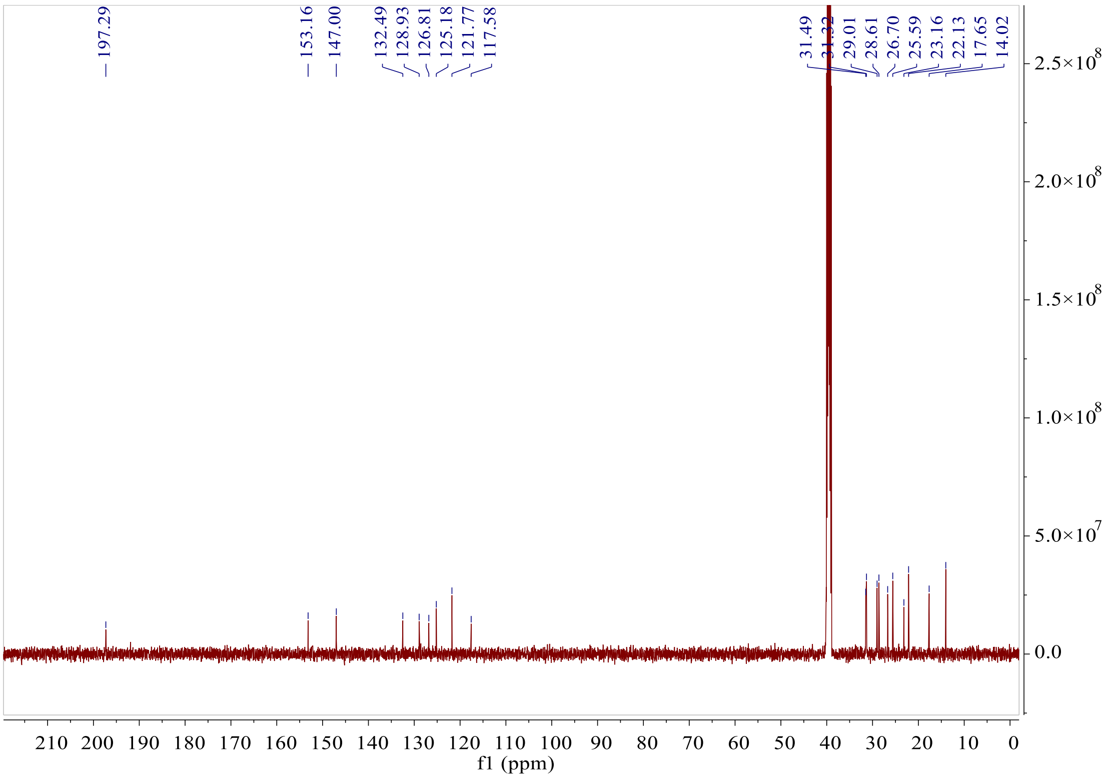

Figure S13. [1]C NMR spectrum of **3** ((DMSO-d_6, 125MHz).

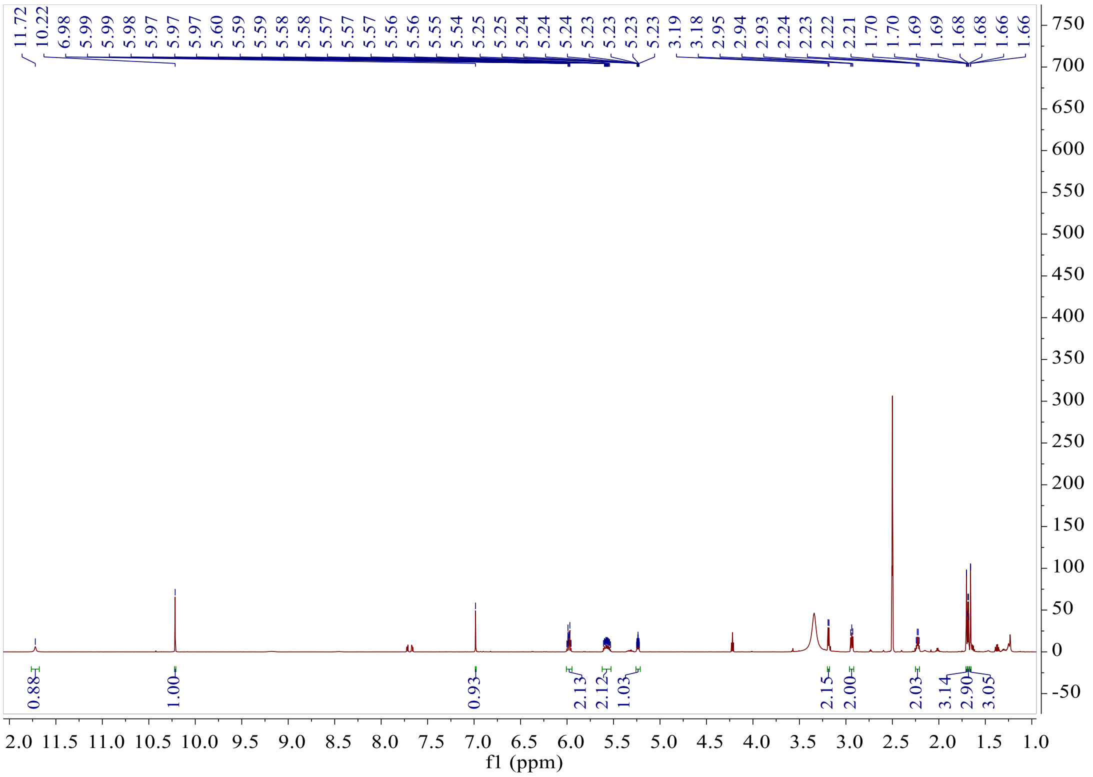

Figure S14. ^{1}H NMR spectrum of **4** (DMSO-d_6, 700 MHz).

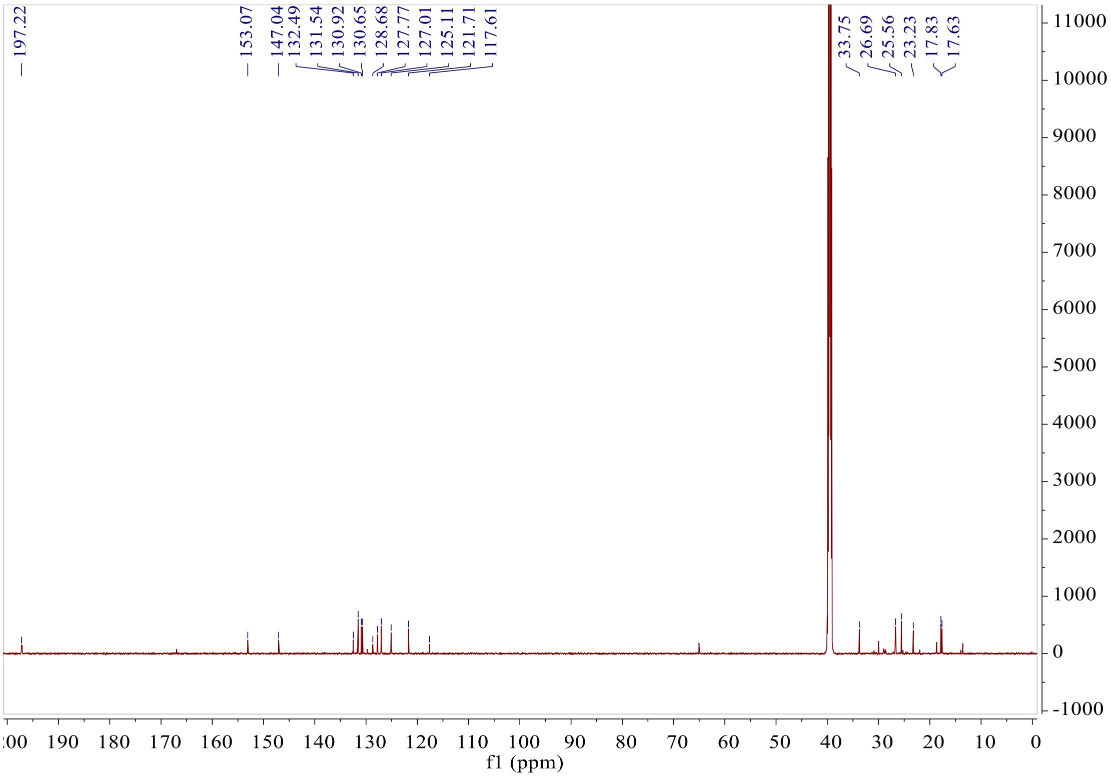

Figure S15. ^{1}C NMR spectrum of **4** (DMSO-d_6, 175 MHz).

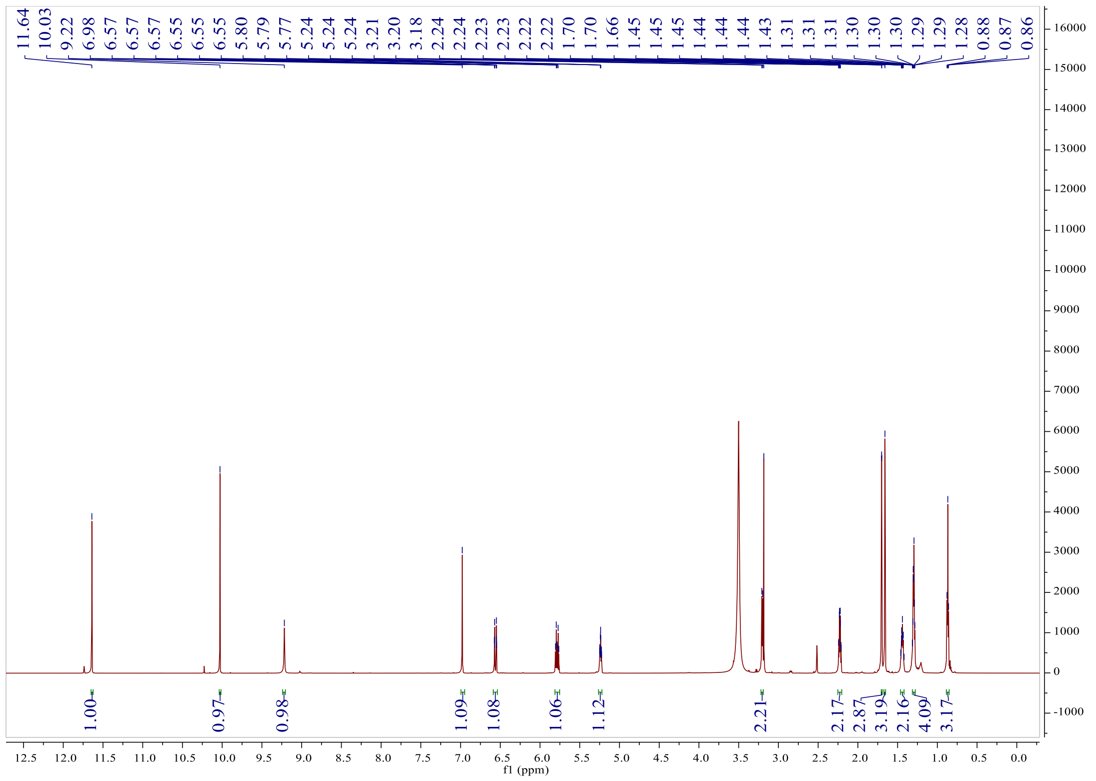

Figure S16. ^{1}H NMR spectrum of **5** (DMSO-d_6, 700 MHz).

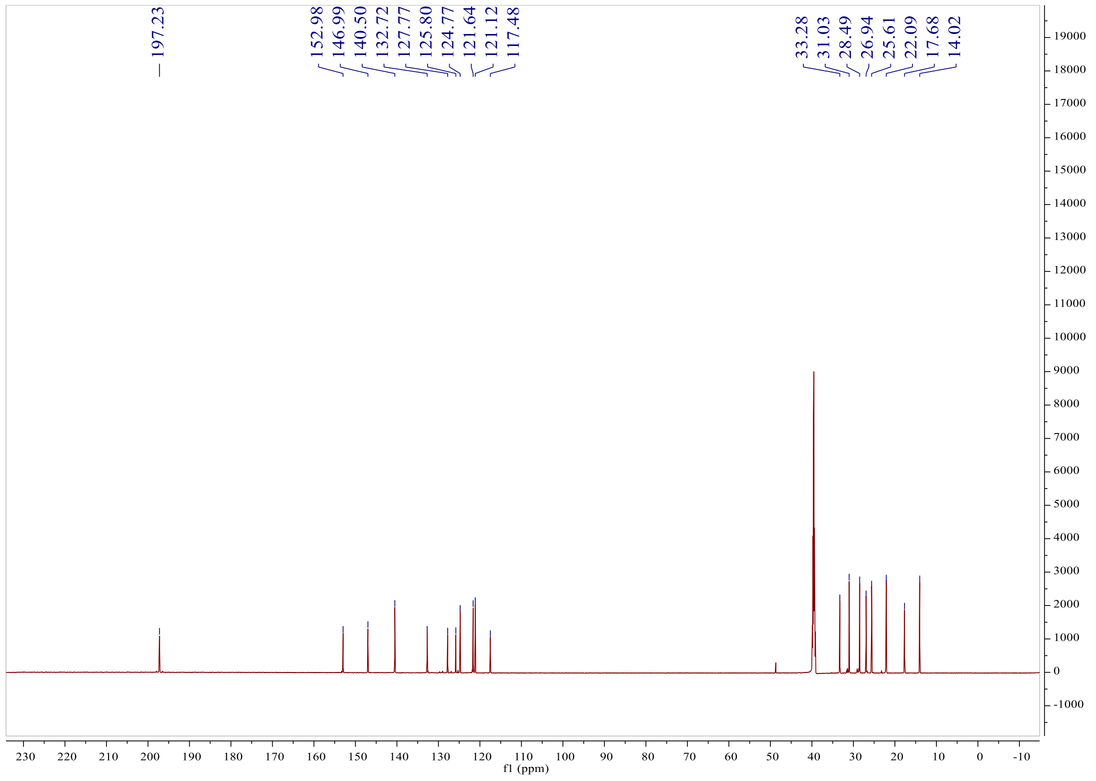

Figure S17. ^{1}C NMR spectrum of **5** (DMSO-d_6, 175 MHz).

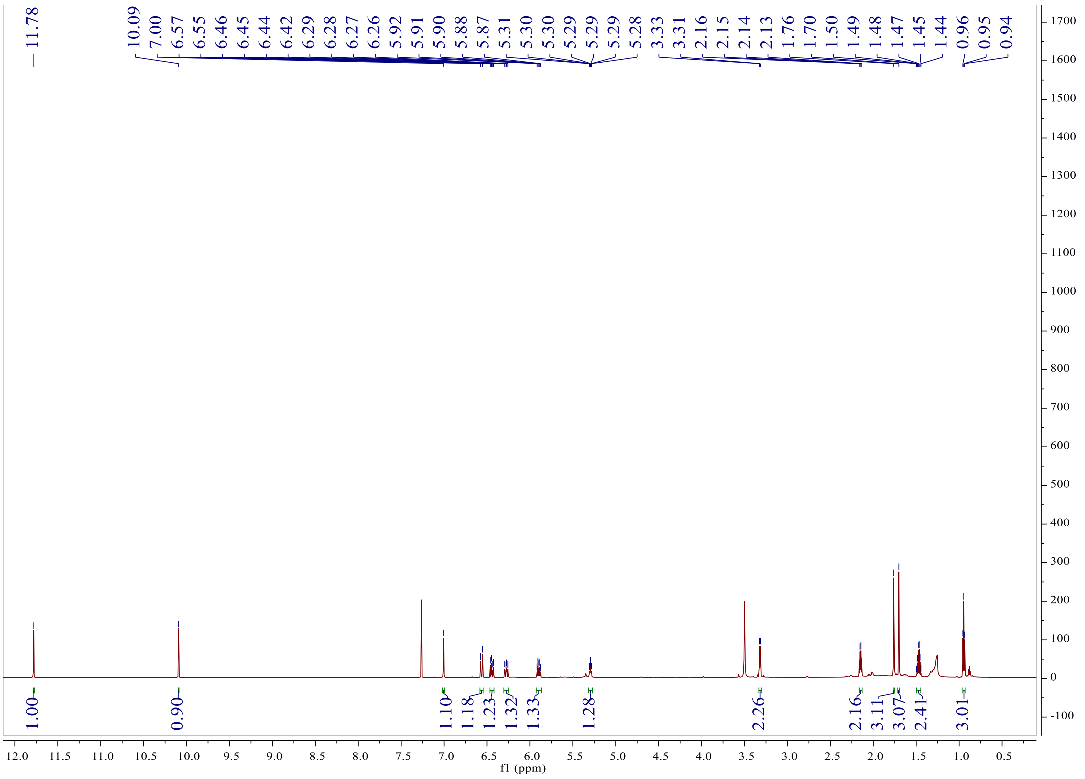

Figure S18. ^{1}H NMR spectrum of **6** ($CDCl_3$, 700 MHz).

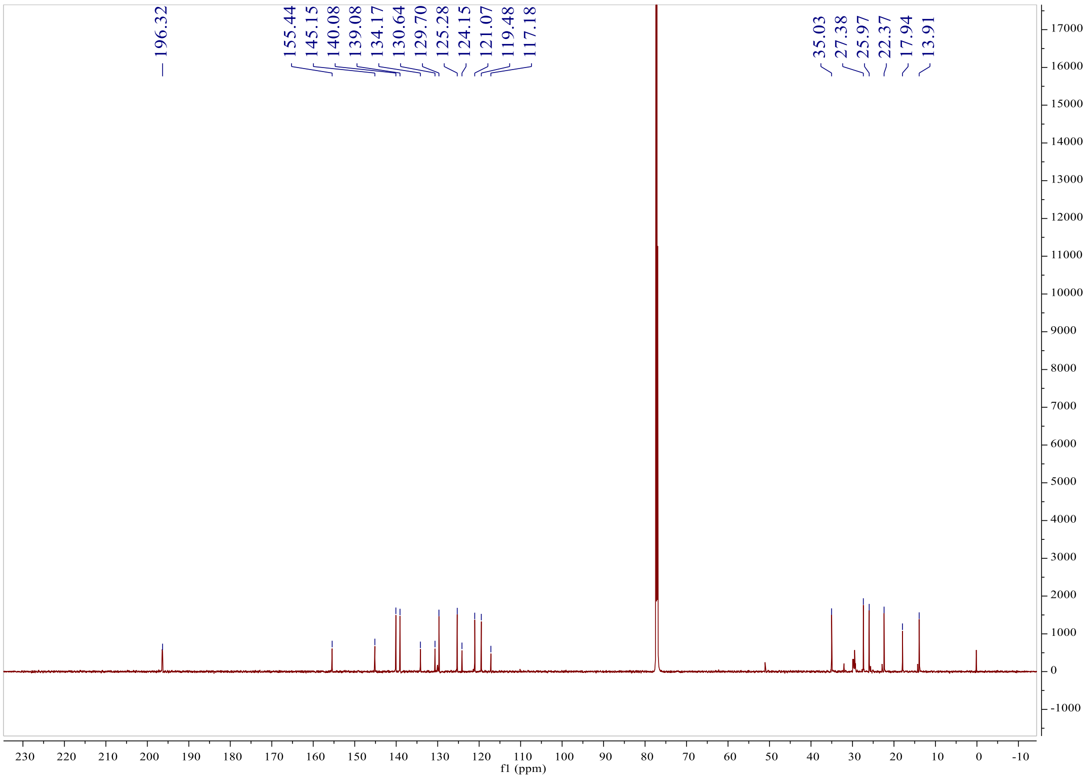

Figure S19. ^{1}C NMR spectrum of **6** $CDCl_3$, 175 MHz).

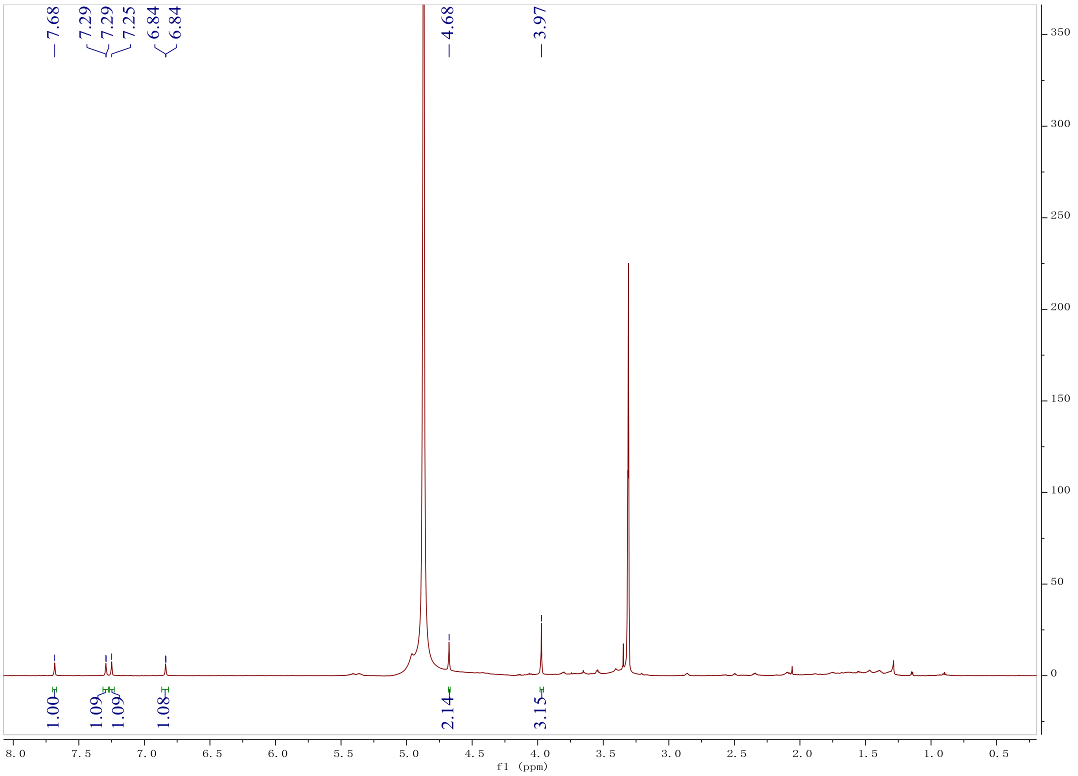

Figure S20. ^{1}H NMR spectrum of **7** (CD_3OD, 700 MHz).

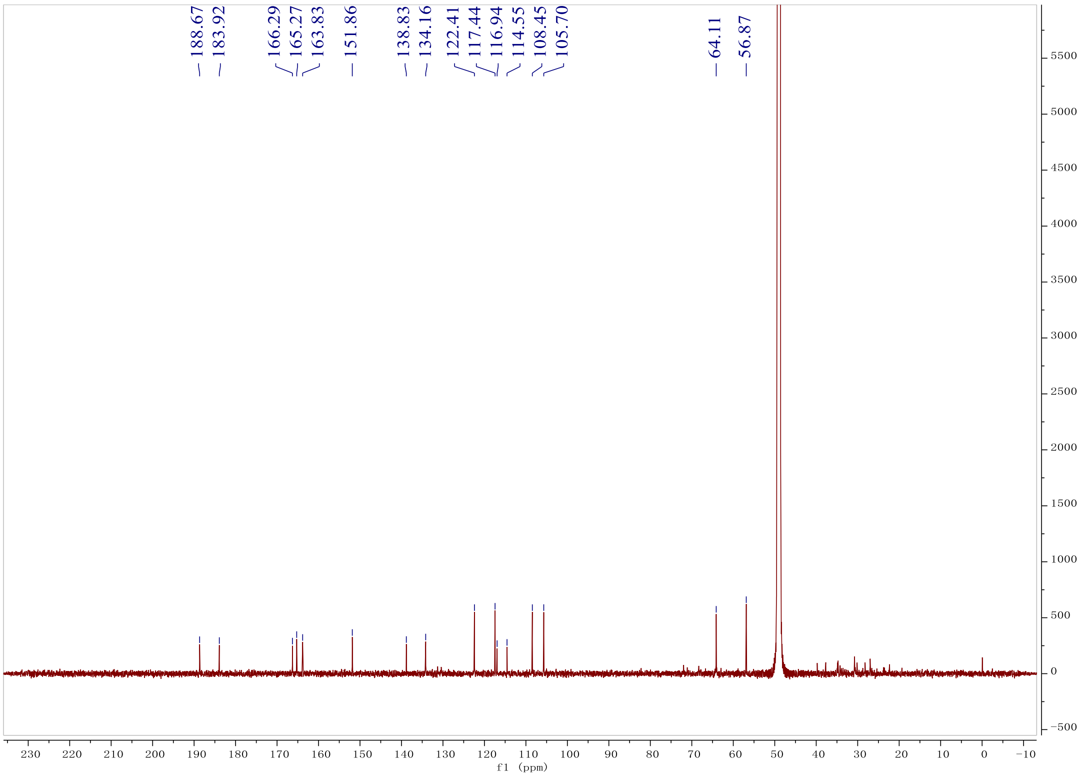

Figure S21. [1]C NMR spectrum of **7** (CD_3OD, 175 MHz).

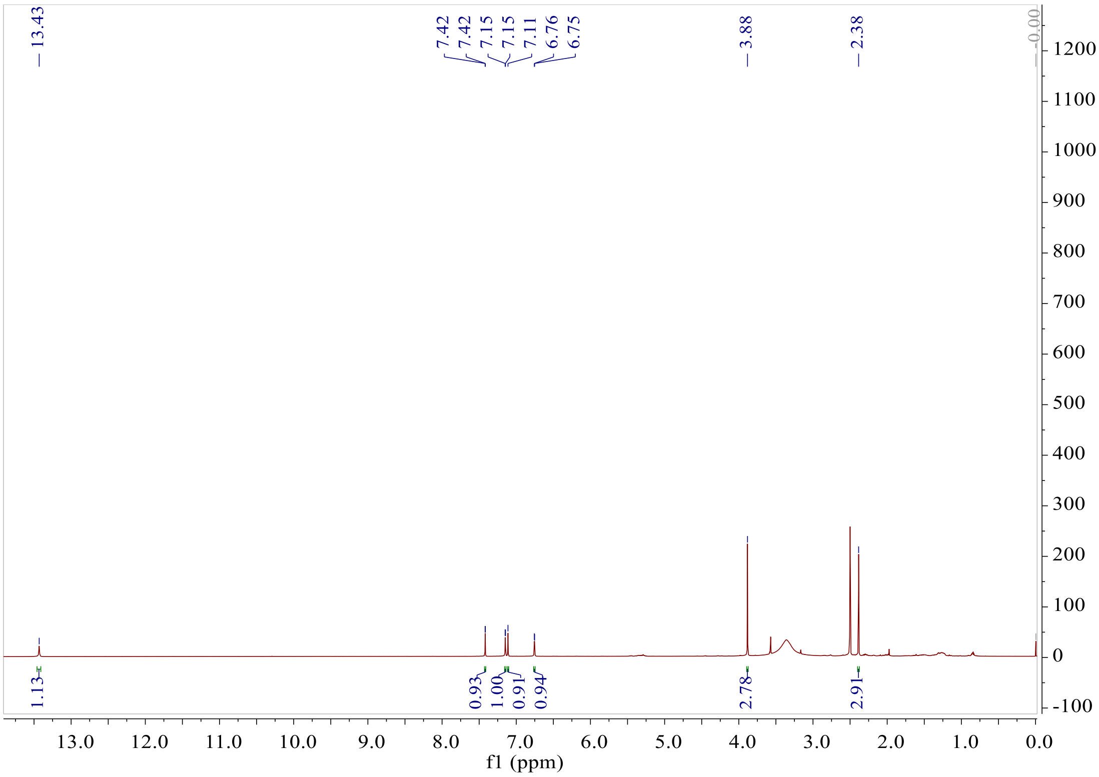

Figure S22. ^{1}H NMR spectrum of **8** (DMSO-d_6, 700 MHz).

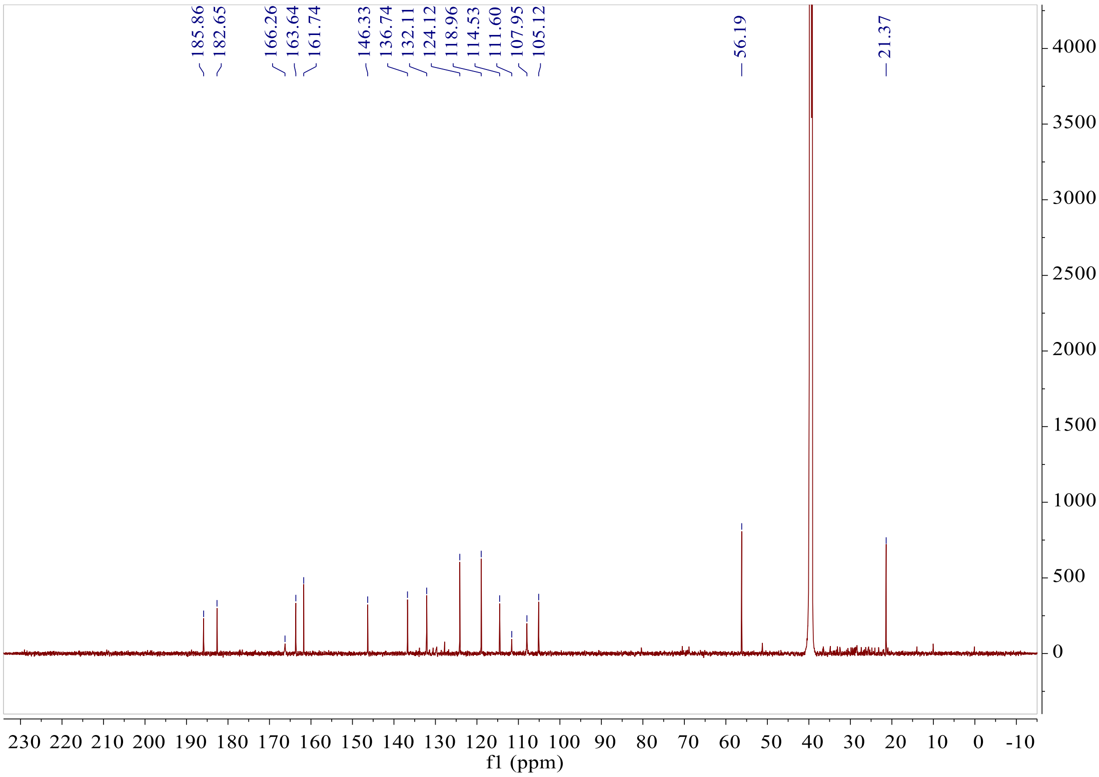

Figure S23. ^{1}C NMR spectrum of **8** (DMSO-d_6, 175 MHz).

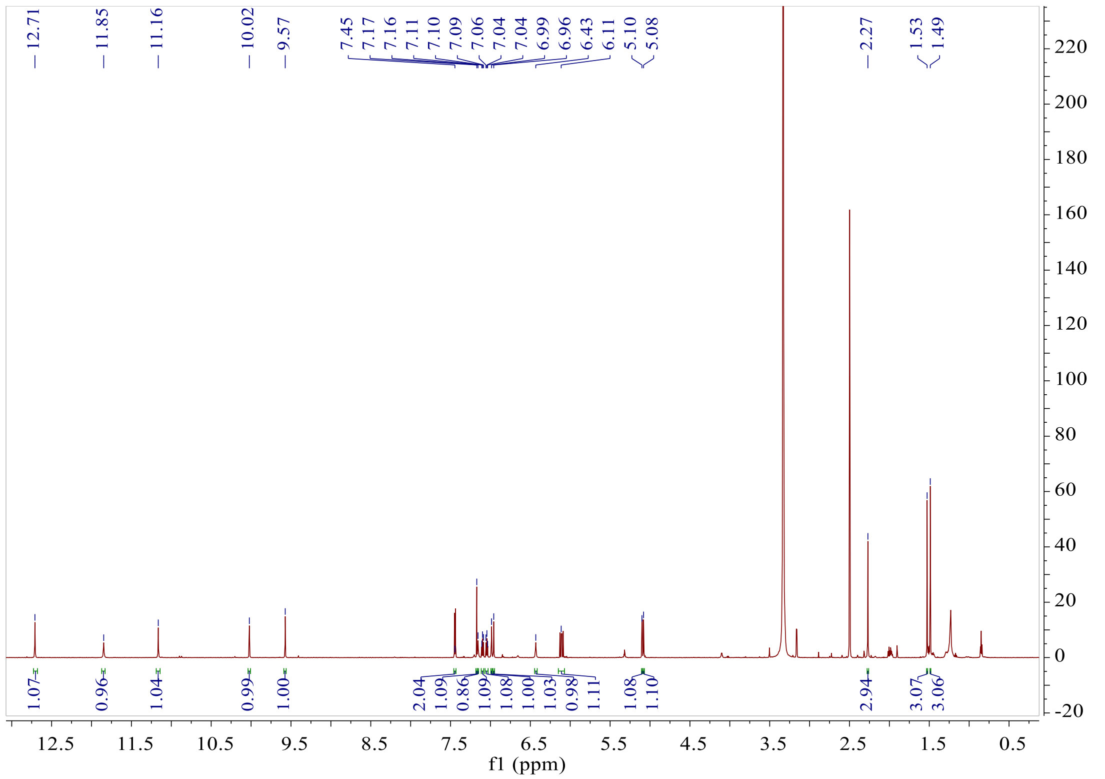

Figure S24. ^{1}H NMR spectrum of **9** (DMSO-d_6, 700 MHz).

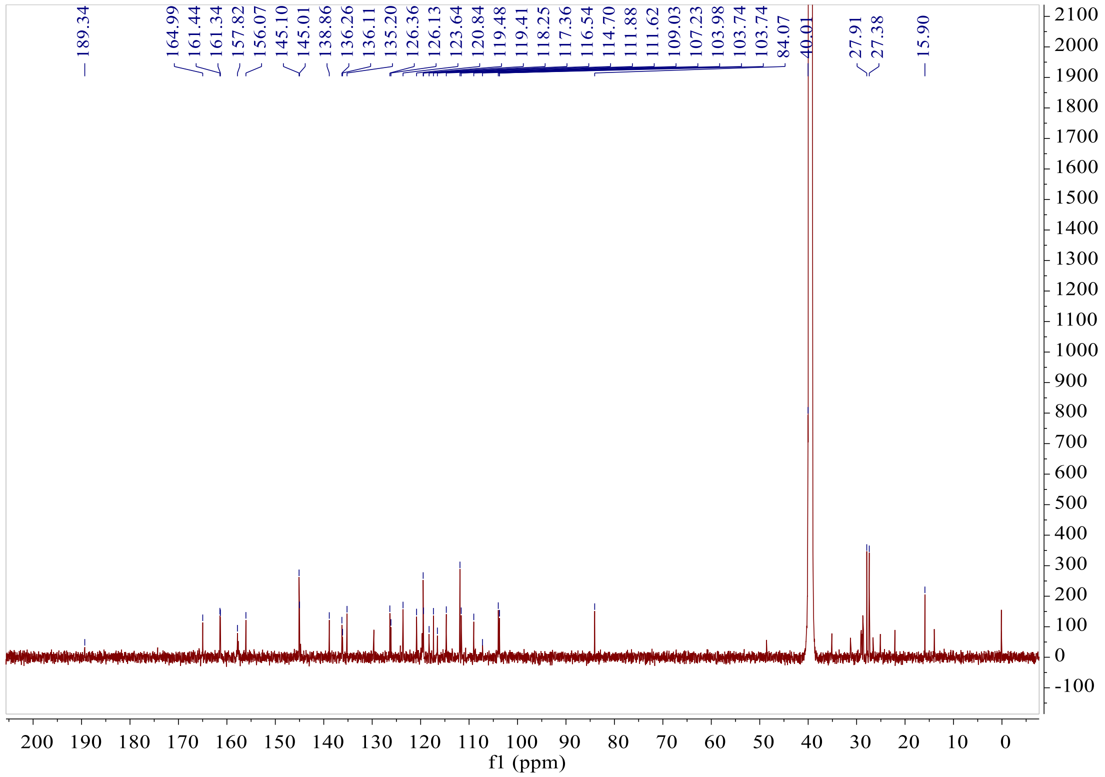

Figure S25. [1]C NMR spectrum of **9** (DMSO-d_6, 175 MHz).

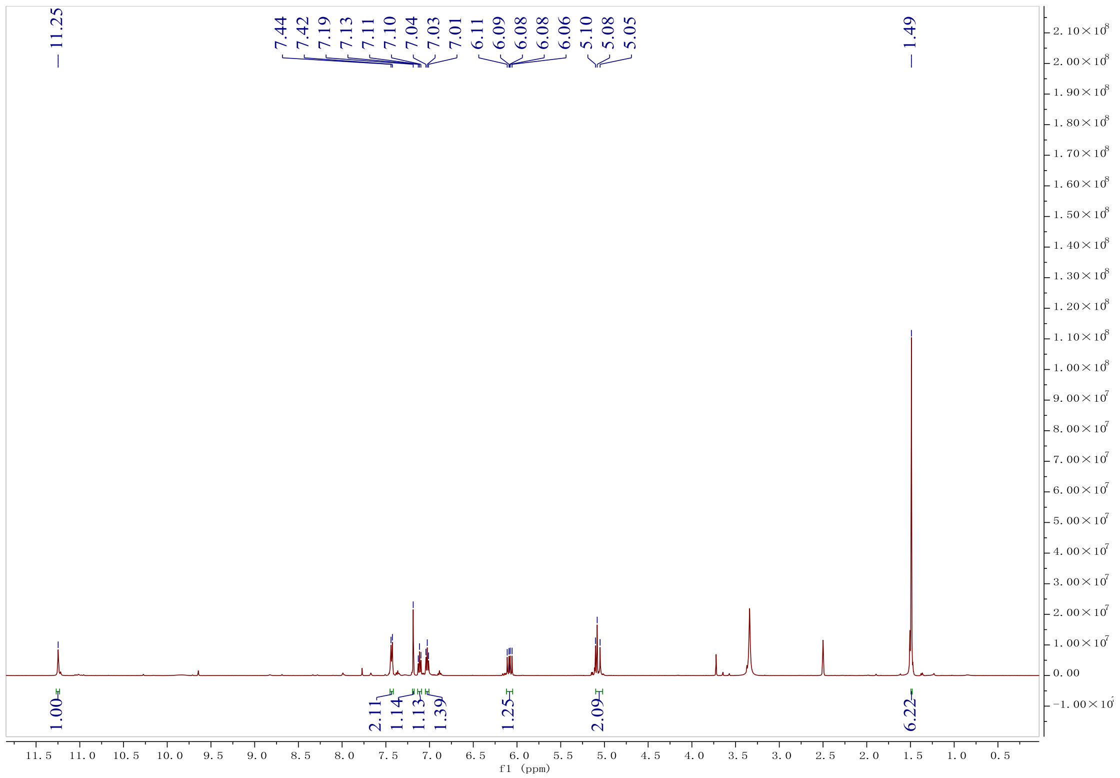

Figure S26. ^{1}H NMR spectrum of **10** (DMSO-d_6, 500 MHz).

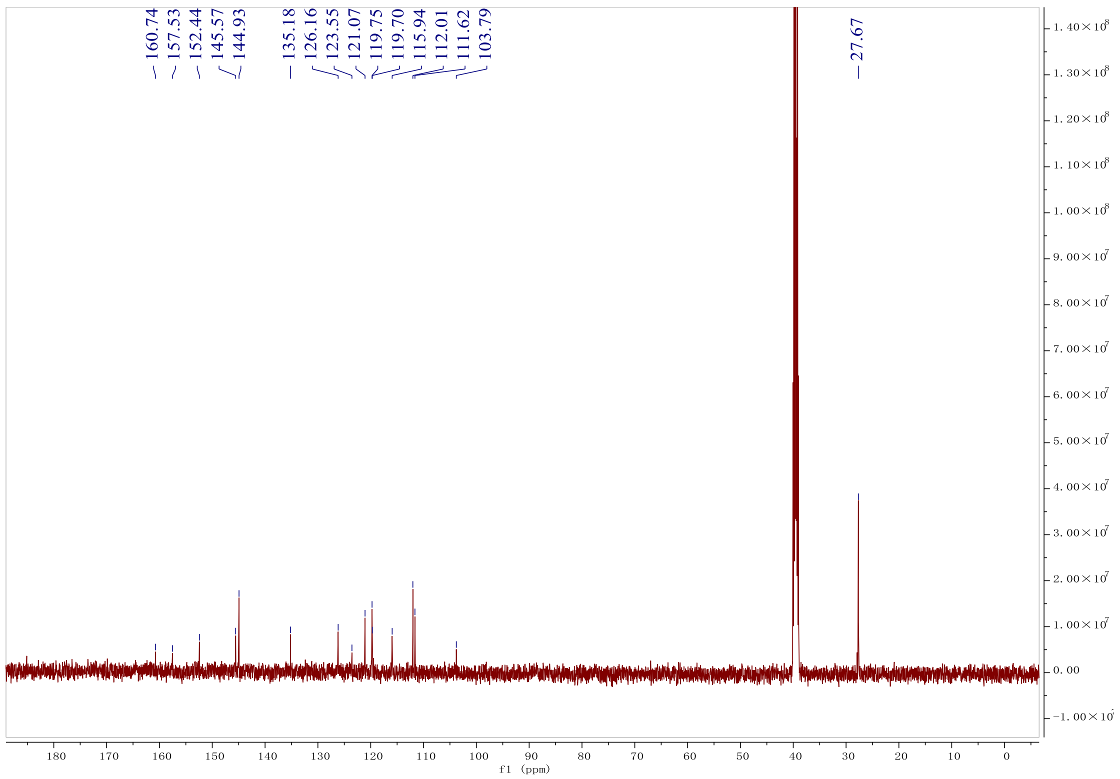

Figure S27. ^{1}C NMR spectrum of **10** (DMSO-d_6, 125 MHz).

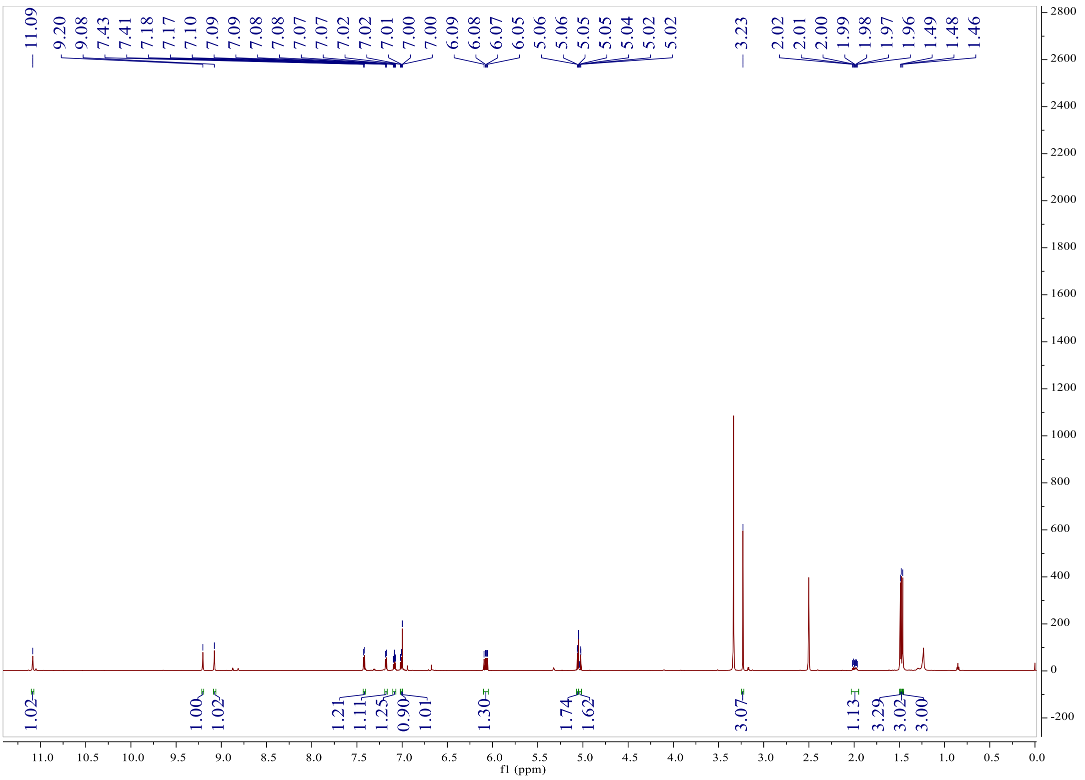

Figure S28. ^{1}H NMR spectrum of **11** (DMSO-d_6, 700 MHz).

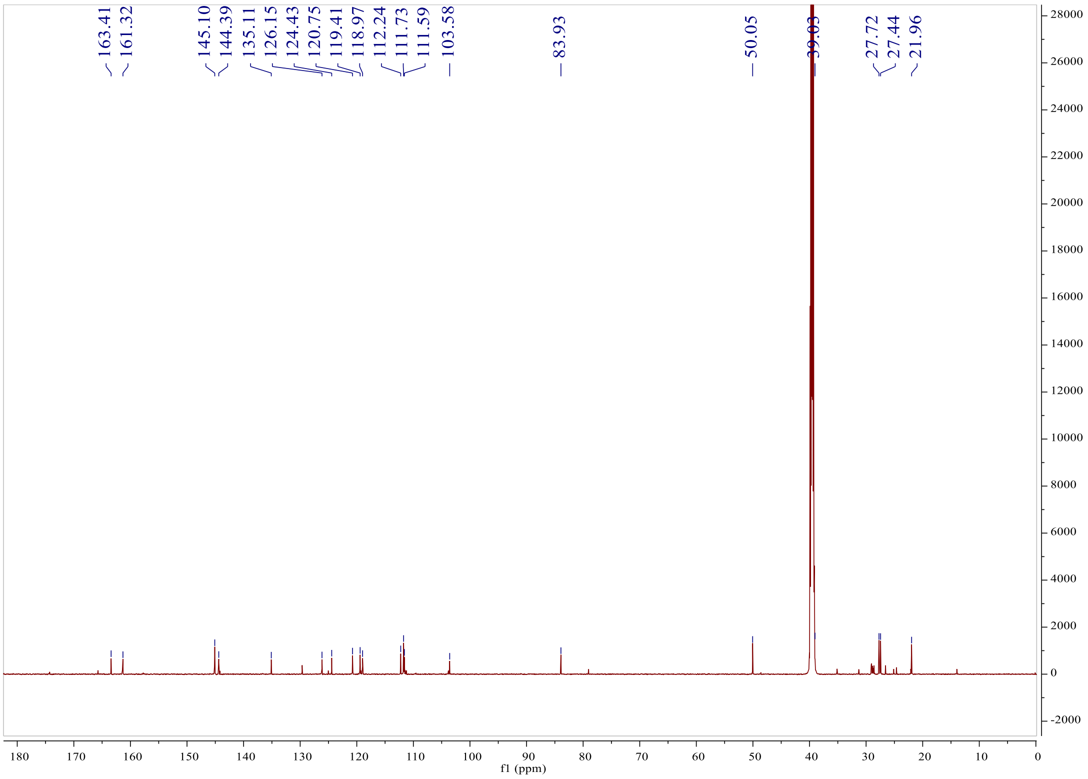

Figure S29. ^{1}C NMR spectrum of **11** (DMSO-d_6, 175 MHz).

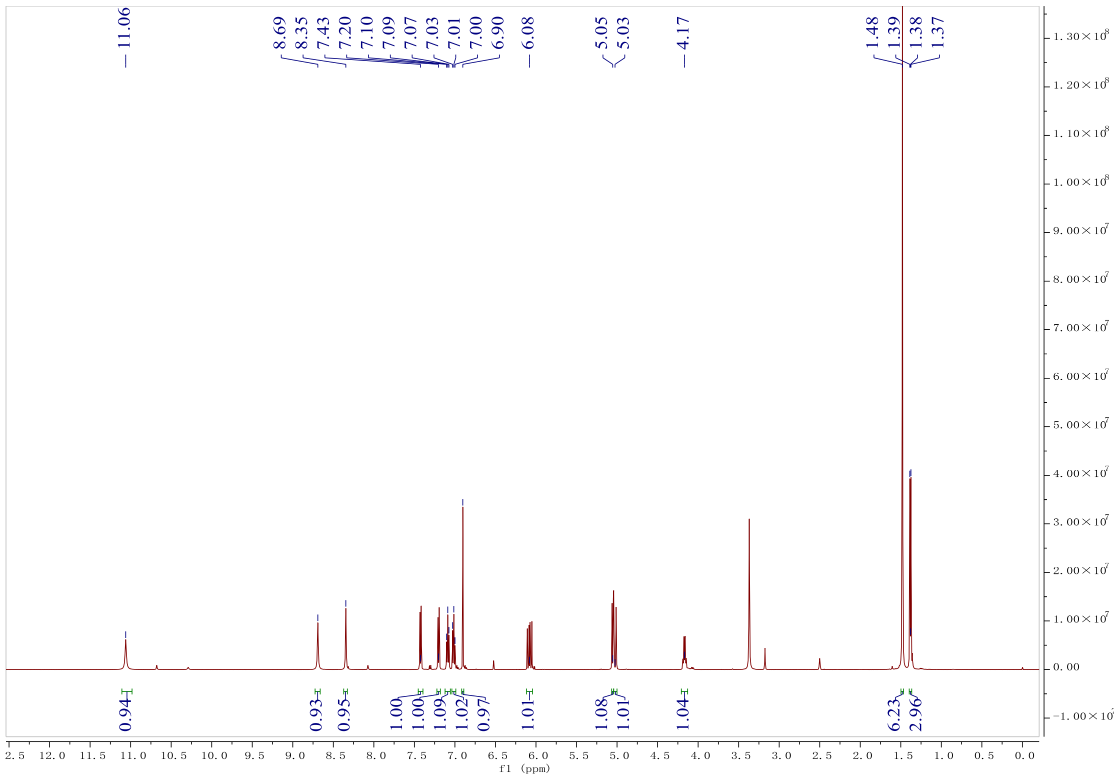

Figure S30. ^{1}H NMR spectrum of **12** (DMSO-d_6, 500 MHz).

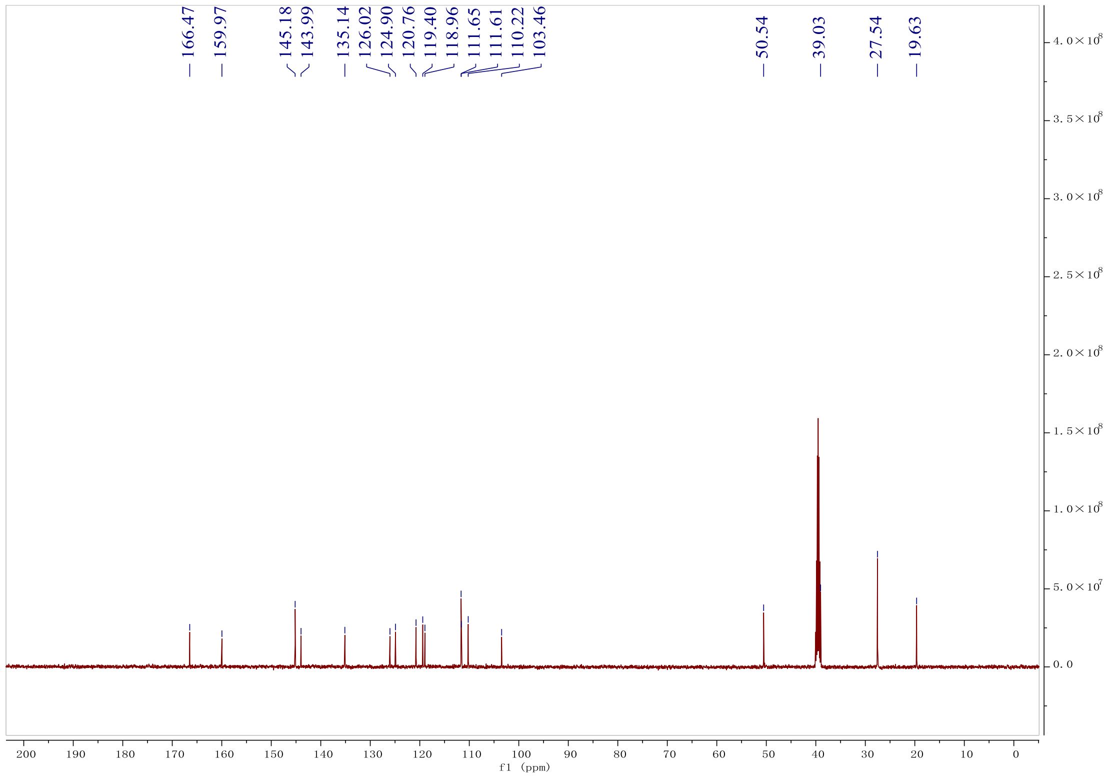

Figure S31. ^{1}C NMR spectrum of **12** (DMSO-d_6, 125 MHz).

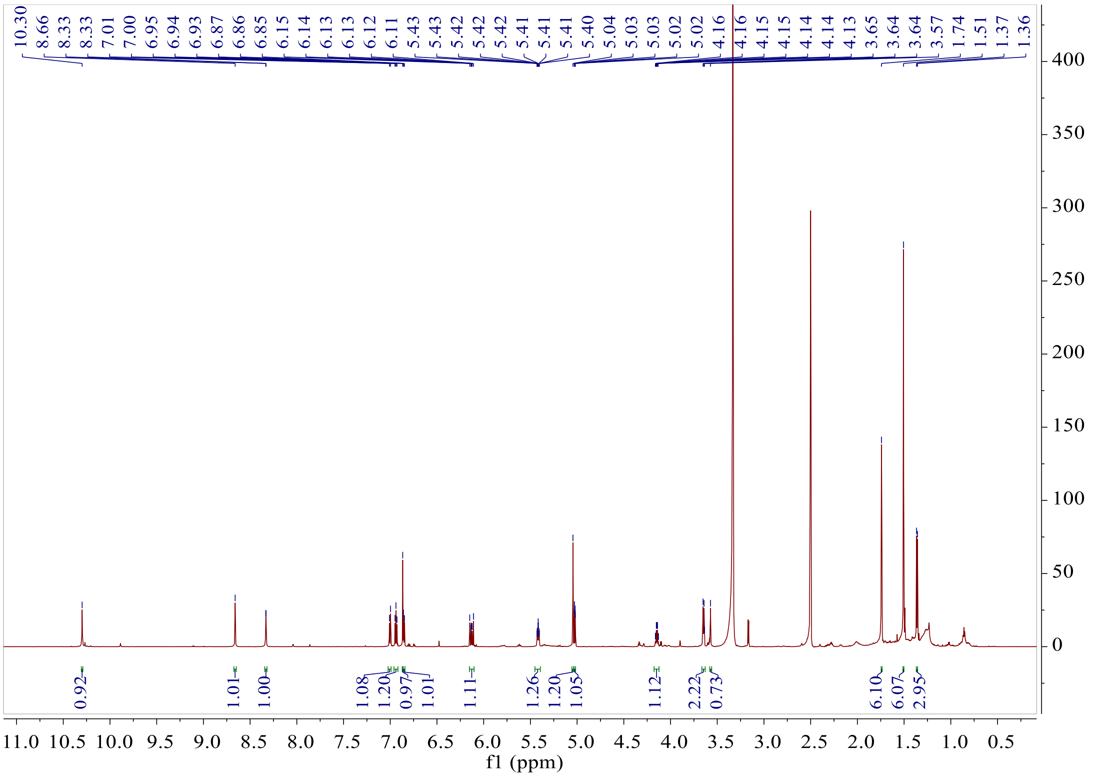

Figure S32. ^{1}H NMR spectrum of **13** (DMSO-d_6, 700 MHz).

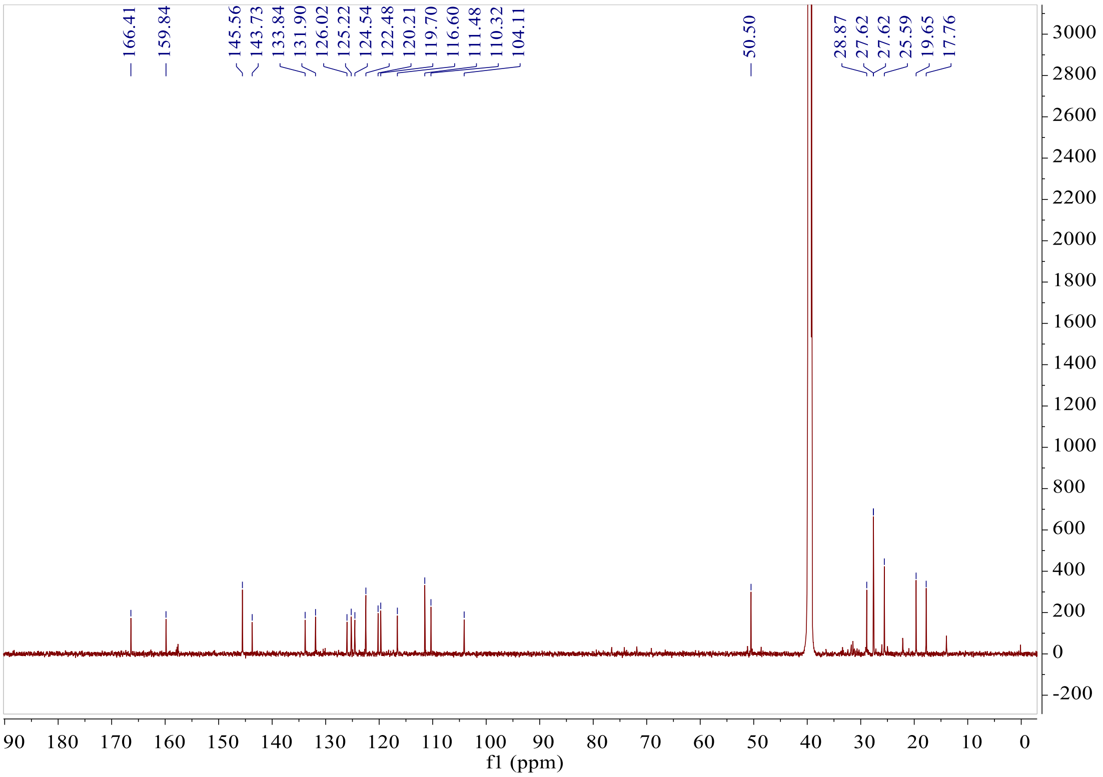

Figure S33. ^{1}C NMR spectrum of **13** (DMSO-d_6, 175 MHz).

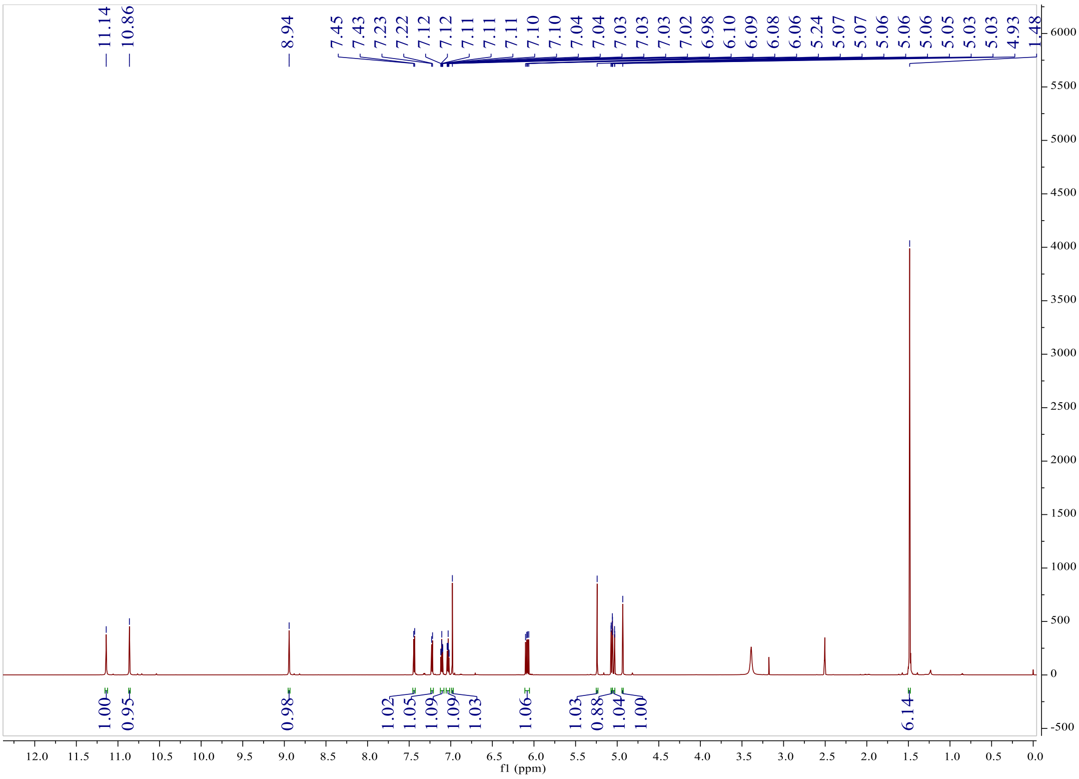

Figure S34. ^{1}H NMR spectrum of **14** (DMSO-d_6, 700 MHz).

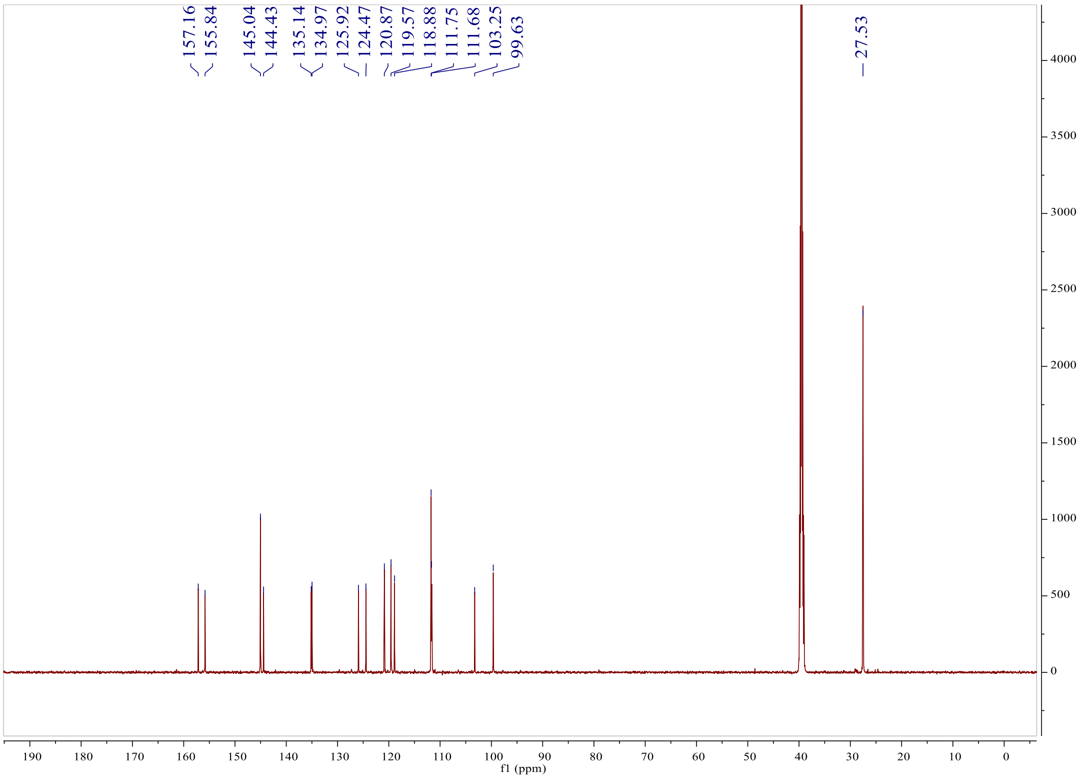

Figure S35. ^{1}C NMR spectrum of **14** (DMSO-d_6, 175 MHz).

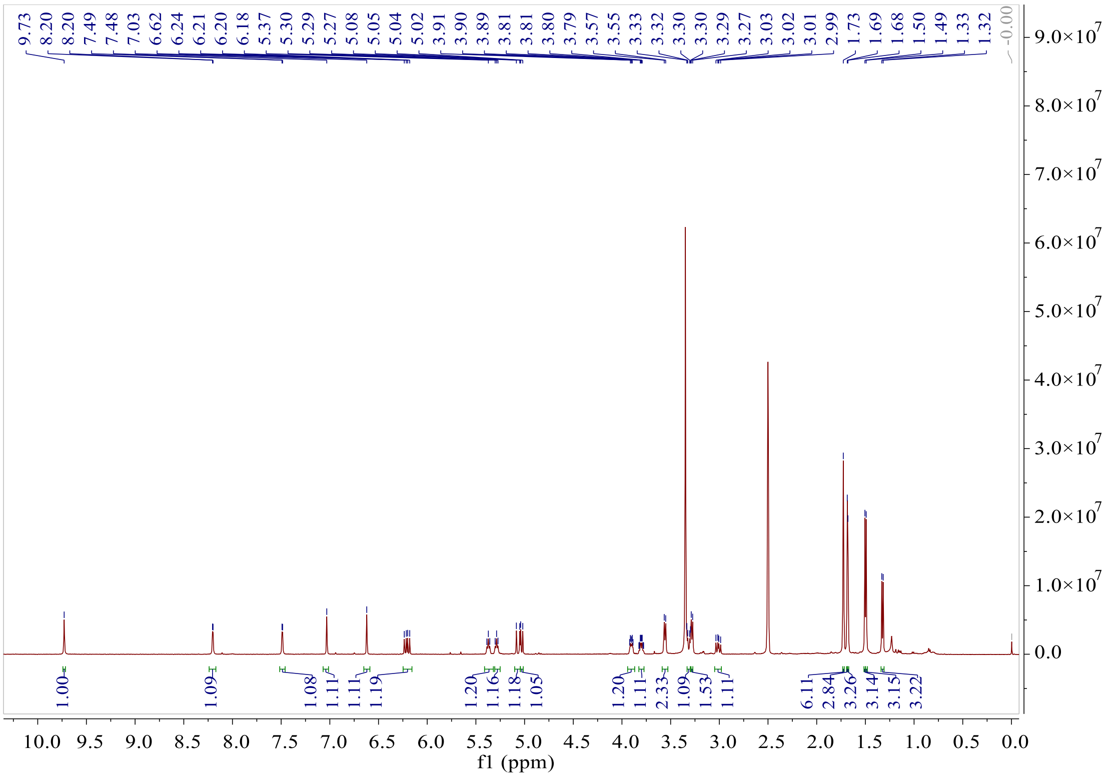

Figure S36. ^{1}H NMR spectrum of **15** (DMSO-d_6, 500 MHz).

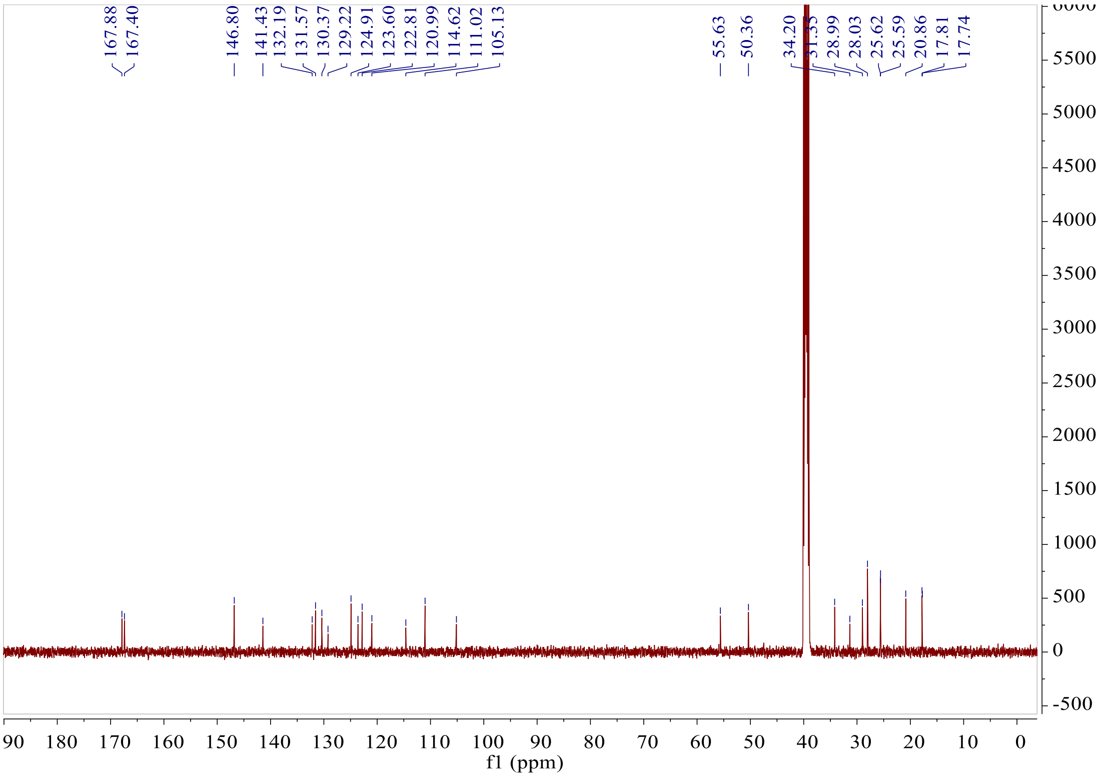

Figure S37. ^{1}C NMR spectrum of **15** (DMSO-d_6, 125 MHz).

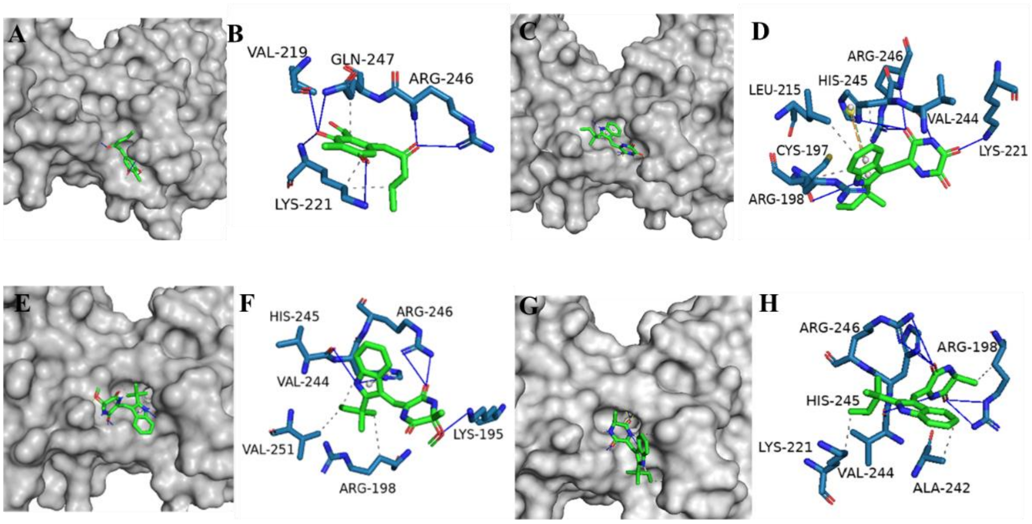

Figure S38. Molecular docking of **1**, **10**, **11**, and **12** with NF-*κ*B p65. Binding sites of **1** (A), **10** (C), **11** (E), and **12** (G) with NF-*κ*B p65. 3D diagram of the binding interactions of **1** (B), **10** (D), **11** (F), and **12** (H) with the active site residues of the NF-*κ*B p65 receptor. Blue solid line: hydrogen bond; grey dotted line: hydrophobic interaction; green dotted line: *π*-*π* stacking interaction; orange dotted line: *π*-cation interaction.

ITS sequence of the strain *Aspergillius* sp. SCSIO41407.

GTACCCTGTTGCTTCGGCGTGGCCACGGCCCGCCGGAGACTAACATTTGAACGCTGTCT
GAAGTTTGCAGTCTGAGTTTTTAGTTAAACAATCGTTAAAACTTTCAACAACGGATCTC
TTGGTTCCGGCATCGATGAAGAACGCAGCGAAATGCGATAATTAATGTGAATTGCAGAA
TTCAGTGAATCATCGAGTCTTTGAACGCACATTGCGCCCCCTGGTATTCCGGGGGGCAT
GCCTGTCCGAGCGTCATTGCTGCCCTCAAGCACGGCTTGTGTGTTGGGCTTCCGTCCCT
GGCAACGGGGACGGGCCCAAAAGGCAGTGGCGGCACCATGTCTGGTCCTCGAGCGTAT
GGGGCTTTGTCACCCGCTCCCGTAGGTCCAGCTGGCAGCTAGCCTCGCAACCAATCTTT
TTAACCAGGTTGACCTCGGATCAGGTAGGGATACCCGCT
